# Mapping the Direction of Nucleocytoplasmic Transport of Glucocorticoid Receptor (GR) in Live Cells Using Two-Foci Cross-Correlation in Massively Parallel Fluorescence Correlation Spectroscopy (mpFCS)

**DOI:** 10.1021/acs.analchem.3c01427

**Published:** 2023-10-02

**Authors:** Stanko N. Nikolić, Sho Oasa, Aleksandar J. Krmpot, Lars Terenius, Milivoj R. Belić, Rudolf Rigler, Vladana Vukojević

**Affiliations:** † Department of Clinical Neuroscience (CNS), Center for Molecular Medicine (CMM), Karolinska Institute, 17176 Stockholm, Sweden; ‡ Institute of Physics Belgrade, University of Belgrade, 11080 Belgrade, Serbia; § Division of Arts and Sciences, Texas A&M University at Qatar, Doha, Qatar

## Abstract

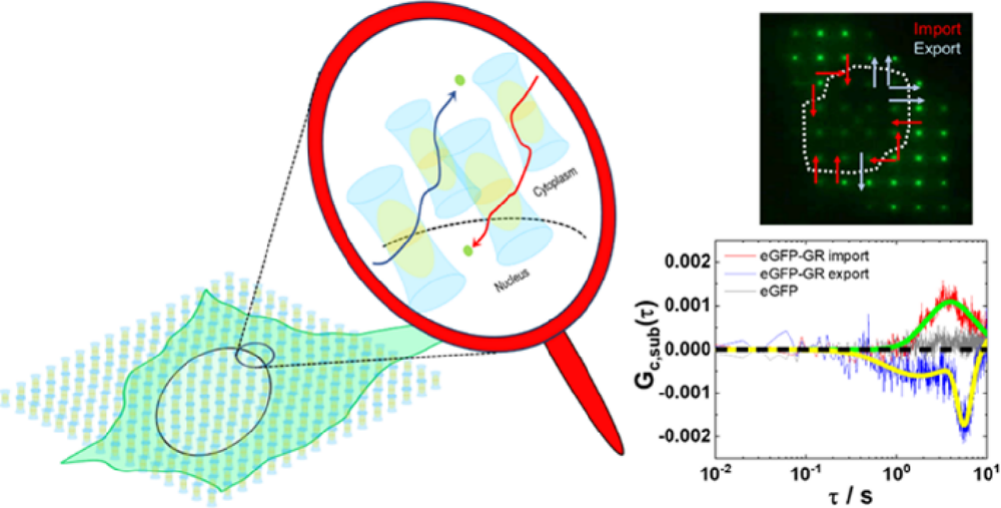

Nucleocytoplasmic transport of transcription factors is vital for normal cellular function, and its breakdown is a major contributing factor in many diseases. The glucocorticoid receptor (GR) is an evolutionarily conserved, ligand-dependent transcription factor that regulates homeostasis and response to stress and is an important target for therapeutics in inflammation and cancer. In unstimulated cells, the GR resides in the cytoplasm bound to other molecules in a large multiprotein complex. Upon stimulation with endogenous or synthetic ligands, GR translocation to the cell nucleus occurs, where the GR regulates the transcription of numerous genes by direct binding to glucocorticoid response elements or by physically associating with other transcription factors. While much is known about molecular mechanisms underlying GR function, the spatial organization of directionality of GR nucleocytoplasmic transport remains less well characterized, and it is not well understood how the bidirectional nucleocytoplasmic flow of GR is coordinated in stimulated cells. Here, we use two-foci cross-correlation in a massively parallel fluorescence correlation spectroscopy (mpFCS) system to map in live cells the directionality of GR translocation at different positions along the nuclear envelope. We show theoretically and experimentally that cross-correlation of signals from two nearby observation volume elements (OVEs) in an mpFCS setup presents a sharp peak when the OVEs are positioned along the trajectory of molecular motion and that the time position of the peak corresponds to the average time of flight of the molecule between the two OVEs. Hence, the direction and velocity of nucleocytoplasmic transport can be determined simultaneously at several locations along the nuclear envelope. We reveal that under ligand-induced GR translocation, nucleocytoplasmic import/export of GR proceeds simultaneously but at different locations in the cell nucleus. Our data show that mpFCS can characterize in detail the heterogeneity of directional nucleocytoplasmic transport in a live cell and may be invaluable for studies aiming to understand how the bidirectional flow of macromolecules through the nuclear pore complex (NPC) is coordinated to avoid intranuclear transcription factor accretion/abatement.

The concentration of transcription factors in the cell nucleus is a key determinant of the kinetics of gene transcription, through which cell identity and function are eventually conferred.^1,2^ It is dynamically controlled in live cells by means of transcription factor import/export into/out of the cell nucleus via the nuclear pore complex (NPC), which is accomplished through two basic mechanisms: passive diffusion and active directional transport.^3,4^ While our understanding of the energetics of the nucleocytoplasmic transport and the biochemical composition and overall organization of the NPC, which is the sole bidirectional gateway through which molecules pass in/out of the cell nucleus, is continuously improving,^5,6^ the spatial organization of directionality of nucleocytoplasmic transport in a single cell remains less well characterized and it is not well understood how the bidirectional nucleocytoplasmic flow of macromolecules is coordinated. The main obstacle to progress is the limited number of analytical methods that can quantitatively characterize the directionality of nucleocytoplasmic transport in live cells at multiple spots simultaneously.

To date, time-resolved fluorescence microscopy imaging and correlation spectroscopy techniques have been shown to be well suited to address this problem in live cells. For example, fluorescence recovery after photobleaching (FRAP), Förster resonance energy transfer (FRET), single-molecule imaging/single particle tracking microscopy, and other related methods have been indispensable for this purpose, as they made it possible to quantify the dynamics of nucleocytoplasmic shuttling and characterize intranuclear reactions and retention.^7−13^ Fluorescence correlation spectroscopy (FCS) and FCS-based methods have enabled us to simultaneously measure with single-molecule and particle sensitivity local translational diffusion coefficients and the local velocities and directions of transport. The value of conventional single-beam FCS for flow measurements was realized already at the inception of FCS when the theoretical background was developed, and a first successful application was demonstrated.^14^ Subsequent studies have shown that conventional FCS is suitable even for more complex applications, such as hydrodynamic flow profiling in microchannel structures,^15^ that the dynamic range of flow velocities that can be measured by FCS is wide, ranging over 4 orders of magnitude,^16^ and that FCS is suitable for involved applications using microfluidic devices^17^ or to characterize cerebral blood flow.^18^ However, these studies have also revealed the limitations of conventional FCS for characterizing molecular transport, most notably the fact that it cannot determine the direction of flow. Hence, dual-focus FCS was developed to overcome this limitation and used to measure the velocity and direction of flow by cross-correlation analysis of signals in two foci.^19,20^ In addition, more specialized FCS-based methods have also developed. For example, interferometric fluorescence cross-correlation spectroscopy (iFCCS) was applied to quantitatively characterize flow and diffusion transport in 2D and 3D;^21^ and multipoint holographic FCS (MP-hFCS), where a spatial light modulator (SLM) was used to generate 8 independent foci, was applied in live cells to quantitatively characterize the nuclear import of the glucocorticoid (GR) receptor via cross-correlation analysis between two independent foci.^22^ While these approaches were invaluable for measuring the velocity and direction of molecular transport, they provided this information for a limited region only. To overcome the limited overview and characterize molecular transport in a wider area, methods that use spatiotemporal correlation analysis of fluorescence fluctuations within an image/series of images were developed, such as two-photon image correlation and cross-correlation spectroscopy,^23^ spatiotemporal image correlation spectroscopy (STICS),^24^ and spatiotemporal image correlation of structured illumination microscopy data.^25^ In addition, imaging FCS methods, such as imaging total internal reflection (ITIR)-FCS^26^ and single-plane illumination microscopy-based FCS (SPIM-FCS),^27,28^ were developed and used to characterize heterogeneity in diffusion and the direction and velocity of transport in multiple positions simultaneously. However, these methods also have some drawbacks that limit their suitability for characterizing the spatial heterogeneity of diffusion and the velocity and direction of transport in live cells. For example, the accuracy and precision of STICS-based methods depends, in addition to the dependence on the concentration and brightness of fluorescent molecules and the point spread function (PSF) of the microscope, on image settings in terms of scanning speed (pixel dwell time, time between the scan lines, and time between images), pixel size, the size of the region of interest (ROI) defined by the number of pixels that it contains, number of frames, and signal acquisition time,^29^ and averaging over a relatively large number of pixels (>64) is needed to allow an accurate characterization of diffusion/motion. This leads to averaging out of spatial heterogeneity of diffusion and the direction and velocity of transport. STICS-based methods also encounter problems when analyzing heterogeneous samples since the presence of bright speckles significantly deforms the autocorrelation curve (ACC). In imaging FCS methods, such as ITIR-FCS and SPIM-FCS, pixel binning and cross-correlation between distant, nonoverlapping pixel areas are necessary to avoid large errors that arise due to crosstalk when cross-correlating the signals from areas that overlap.^26^

We have recently developed massively parallel FCS (mpFCS) in which geometrically independent multiple excitation foci are formed in the specimen and demonstrated its value for quantitative scanning-free confocal fluorescence microscopy imaging and characterization of fast dynamic processes in live cells.^30,31^ Here, we present how this new FCS-based imaging modality can be used to map the spatial organization of directionality of nucleocytoplasmic transport in live cells using, as a model system, live HEK cells expressing the GR fused at the N-terminal end with the enhanced green fluorescent protein (eGFP-GR). The GR is a hormone-dependent receptor that belongs to the nuclear receptor superfamily of transcriptional regulatory factors.^32^ It is indispensable for maintaining homeostasis under normal physiology and under stress. In unstimulated cells, the GR resides in the cytoplasm, bound into a large multiprotein complex.^33,34^ Upon stimulation with endogenous or synthetic ligands^35^ and their binding to the cytoplasmic GR, ligand-induced GR translocation to the cell nucleus occurs. In the cell nucleus, GR regulates the transcription of numerous genes—it is estimated that glucocorticoid-responsive genes probably represent 3–10% of the human genome,^36^ acting either as an activator or repressor of genes across the genome by direct binding of GR oligomeric forms (dimers or tetramers^37−42^) to glucocorticoid response elements (GREs) on the genome DNA and/or by physically associating with other transcription factors (e.g., NF-κB). The GR concentration in the cell nucleus is dynamically regulated through a complex, perpetually ongoing bidirectional nucleocytoplasmic shuttling of GR.^43^ Despite being extensively studied,^10,11,44,45^ little is known about how bidirectional flow of GR is spatially organized and coordinated in a live cell to avoid collision of inward/outward shuttling GR and its intranuclear accretion/abatement.

## Experimental Section

### Cell Culture and Pharmacological Treatment

HEK cells (ATCC) were grown in 25 mL cell culture flasks with filter caps (T-25, Sarstedt) in Dulbecco’s modified Eagle medium (DMEM; Gibco) supplemented with 10% fetal bovine serum (FBS; Gibco) and 1% penicillin–streptomycin (Gibco), 100 U/mL final concentration penicillin, and 100 μg/mL streptomycin and maintained in a humidified atmosphere containing 5% CO_2_ at 37 °C.

One day before transfection, HEK cells were seeded in Lab-Tek 8-well chambered coverglass (Thermo Fisher Scientific), with a seeding density of 1.0 × 10^4^ cells per well (volume of 400 μL). The cells were transfected with 100 ng of peGFP-C1 or peGFP-GR-C1 plasmids for expression of the enhanced green fluorescent protein (eGFP) or wild-type human GR α fused with eGFP, respectively, using 0.2 μL of Lipofectamine 2000 (Thermo Fisher Scientific). Twenty-four hours after transfection, the cell culture medium was replaced as described below, and the cells were subjected to further analysis.

To induce GR translocation into the nucleus, the synthetic ligand dexamethasone (Dex; Sigma-Aldrich) was used. For pharmacological treatment, a 2 mM Dex stock solution prepared by dissolving Dex in dimethyl sulfoxide (DMSO) was diluted to 500 nM in phenol red-free medium FluoroBrite DMEM (Gibco) and the cell culture medium was replaced. In control experiments, the phenol red-free medium FluoroBrite DMEM (Gibco) was used.

### Chemicals

Yellow-green fluorescence (Ex/Em: 505/515) carboxylate-modified polystyrene nano/microspheres of different nominal diameters, *d* = 100 nm (*D*_fs,100_ = 4.4 × 10^–12^ m^2^ s^–1^) or 2.0 μm (FluoSpheres Size Kit #2), and carboxylate-functionalized quantum dot nanocrystals, *d* = 20 nm, with emission maxima at 525 nm (Qdot 525 ITK Carboxyl Quantum Dots; Molecular Probes, Life Technologies Corporation, USA) were used for mpFCS instrument calibration and performance characterization. Thirty min of sonication was applied to minimize the agglomeration of quantum dots/fluospheres.

For fFMI instrument alignment, a uniform thin layer of Rh6G was prepared by squeezing 1 μL of concentrated Rh6G solution in water between a microscopic slide and a cover glass (no. 1.5 thickness, 22 × 40 mm) and allowed to dry.

### Confocal Laser Scanning Microscopy (CLSM) Imaging

Time-lapse CLSM imaging was performed using the LSM880 system (Carl Zeiss), equipped with the VIS-laser module comprising the Ar-ion laser (458, 488, and 514 nm), C-Apochromat 40×/1.2 N.A. W objective, and gallium arsenide phosphide (GaAsP) detector. eGFP was excited using a 488 nm Ar-ion laser line. Fluorescence was detected in the 493–630 nm range. Following treatment with 500 nM Dex, time-lapse confocal images were acquired at 1 min intervals for 35 min.

### Optical Setup for Massively Parallel Fluorescence Correlation Spectroscopy (mpFCS)

The optical setup for mpFCS is described in detail in our previous work^30,31^ and the Supporting Information (Section SI1, Figure S1). Briefly, the instrument was built using an inverted epi-fluorescence microscope Axio Observer D1 frame equipped with a C-Apochromat 63×/1.2 W Corr objective lens (Carl Zeiss, Germany). A continuous wavelength (CW) 488 nm frequency-doubled diode laser Excelsior 488 (Spectra-Physics, France) was used as the excitation light source. A Diffractive Optical Element (DOE; Holoeye, Germany) was used to precisely create a spot-wise illumination pattern that matches the distribution of single-photon avalanche diodes (SPADs) on the Single Photon Counting SPC2 or the SPC3 camera (Micro Photon Devices MPD, Italy).^46^ To enable fast sample localization, an 18.0 megapixel digital single-lens reflex (DSLR) camera EOS 600D (pixel size of 18.5 μm^2^ and pixel pitch of 4.3 μm; Canon Inc., Japan) was coupled to the side port of the microscope opposite to the SPC2/SPC3 camera, and the light path between the two camera ports was manually switched.

### mpFCS Data Acquisition

For mpFCS data acquisition in aqueous suspension of fluospheres or quantum dots, an instrumental setup comprising a 32 × 32 DOE and the SPC2 camera was used.^30^ Here, the photosensitive area of the chip consists of 32 × 32 circular SPADs that are 20 μm in diameter, and the distance between adjacent diodes along a row/column, i.e., the pitch of the camera, is 100 μm. A total of 131,000 frames were acquired every 20.74 μs, yielding 1024 fluorescence intensity fluctuation traces recorded over 2.71 s. The data was stored in the internal memory of the SPC2 camera during signal acquisition and then transferred to a Dell Precision Fixed Workstation T5600 Xeon E5–2620 2 GHz equipped with an NVIDIA GeForce GTX 780 graphic card containing 2304 Compute Unified Device Architecture (CUDA) for fast data analysis by two-foci cross-correlation as described in Data Processing.

For mpFCS measurements in live cells, the more sensitive SPC3 camera with significantly reduced afterpulsing was used; here, the photosensitive area of the chip consists of 64 × 32 circular SPADs that are 30 μm in diameter, and the distance between adjacent diodes along a row/column, i.e., the pitch of the camera, is 150 μm. In addition, a spot-wise, 16 × 16 illumination was applied and every other SPAD in the centrally positioned 16 × 16 SPADs of the SPC3 camera was used for signal detection.^31^ Fifteen minutes after treatment with 500 nM Dex, 1,048,570 frames were acquired at a temporal resolution of 100 μs/frame, yielding 256 fluorescence intensity fluctuation traces recorded over 104.85 s. During signal acquisition, the data was stored in the internal memory of the SPC3 camera and then transferred to a PC for further analysis using the Origin Data Analysis and Graphing software (OriginLab), as described in Data Processing.

### Data Processing

To calculate the two-foci cross-correlation curve (tfCCC) for arbitrary pixels *a* and *b* of the SPAD camera, the following definition applies:
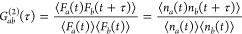
1where *F*_*a*_(*t*) and *F*_*b*_(*t*) are fluorescence intensities that are directly proportional to the number of emitted photons *n*_*a*_(*t*) and *n*_*b*_(*t*) at time *t* for pixels *a* and *b*, respectively, the angular brackets indicate a time average, and τ is the lag time, also called correlation time. However, for the fast, real-time, and highly precise computation of the auto- and cross-correlation functions, a multitau algorithm was developed. Explanation of the multitau procedure can be found in the literature.^31,32,47,48^

For *in vitro* mpFCS measurements in dilute suspensions of fluospheres or quantum dots, the multitau algorithm was used to analyze the raw photon counts directly, and the following formula was used for the calculation of the tfCCCs:
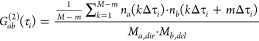
2

Briefly, in eq 2, τ_*i*_ is the lag time and Δτ_*i*_ is the sampling time for channel *i* (*i*th value of the auto- or cross-correlation function). The bin width for the first group is Δτ_1_ = 20.74 μs. Numbers *m* and *M* are integers defined as *m* = τ_*i*_/Δτ_*i*_ and *M* = *T*/Δτ_*i*_, where *T* is the total measurement time. The number of photons counted over a time interval [(*k* – 1)Δτ_*i*_, *k*Δτ_*i*_] is denoted as *n*(*k*Δτ_*i*_). One can see that the correlation analysis boils down to obtaining the sum of the products *n*(*k*Δτ_*i*_)·*n*(*k*Δτ_*i*_ + *m*Δτ_*i*_) of the counted photons at time *k*Δτ_*i*_ and *m*Δτ_*i*_ later. In eq 1, *n*_*a*_(*k*Δτ_*i*_) and *n*_*b*_(*k*Δτ_*i*_) denote photon counts at time *k*Δτ_*i*_ for pixels indicated by the subscript, while *M*_*a*,dir_ and *M*_*b*,del_ are so-called direct and delayed monitor for pixels *a* and *b*, respectively. They are computed using the following equations:
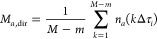
3
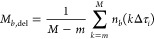
4

It is important to note that *G*_*ab*_^(2)^(τ_*i*_) ≠ *G*_*ba*_^(2)^(τ_*i*_). Thus, for each pair of pixels, there are two tfCCCs that are different in the case of directed molecular/particle motion, hence providing information about the direction of movement. Notably, a two-foci autocorrelation function is obtained when *a* = *b* in eqs 1 and 2.

For measurements in live cells, Origin Data Analysis and Graphing software (OriginLab) was used to compute cross-correlation curves between foci in the cytoplasm and nucleus. As a first step, Origin Data Analysis and Graphing software (OriginLab) was used to post acquisition account for photobleaching in individual time series. To this aim, data compression was first applied by binning the data into 10 ms bins, and a fourth-order polynomial function was used to fit the fluorescence intensity traces that are decaying due to photobleaching:^49,50^
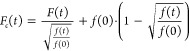
5Here, *F*(*t*) and *F*_*c*_(*t*) are fluorescence intensities of raw and corrected data at time *t*, respectively, and *f*(0) and *f*(*t*) denote values of the fourth-order polynomial function at time 0 and *t*, respectively. We then employed eq 2, substituting raw photon intensities (counts) with the fourth-order polynomial functions from eq 5. The forward, cytoplasm to nucleus, and backward, nucleus to cytoplasm, translocation cross-correlation curves are respectively defined as follows:
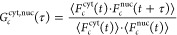
6
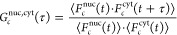
7where *F*_*c*_^nuc^(*t*) and *F*_*c*_^cyt^(*t*) are photobleaching-corrected fluorescence intensity time series recorded in the selected pixels in the nucleus and cytoplasm, respectively.

To readily visualize the translocation direction between two foci, the tfCCC in the direction of nucleus to cytoplasm (*G*_*c*_^nuc,cyt^(τ)) is subtracted from the tfCCC in the direction of cytoplasm to nucleus (*G*_*c*_^cyt,nuc^(τ)):
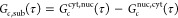
8

In this way, a positive peak in *G*_*c*,sub_(τ) signifies nuclear import, whereas a negative peak indicates nuclear export. We refer to *G*_*c*,sub_(τ) as the subtracted cross-correlation curve.

## Results

### Theoretical Results

Theoretical background and results of numerical simulations of fluorescent fluosphere motion are presented in the Supporting Information (Section SI2, Figures S2–S5).

### Proof-of-Concept Results Using Immobilized q-Dots and a Motorized Stage with Nanometer Positioning Precision

Results of proof-of-concept measurements using a precisely controllable motorized stage to move the sample are presented in the Supporting Information (Section SI3, Figures S6 and S7).

### In Vitro Experimental Results

**Figure 1:**
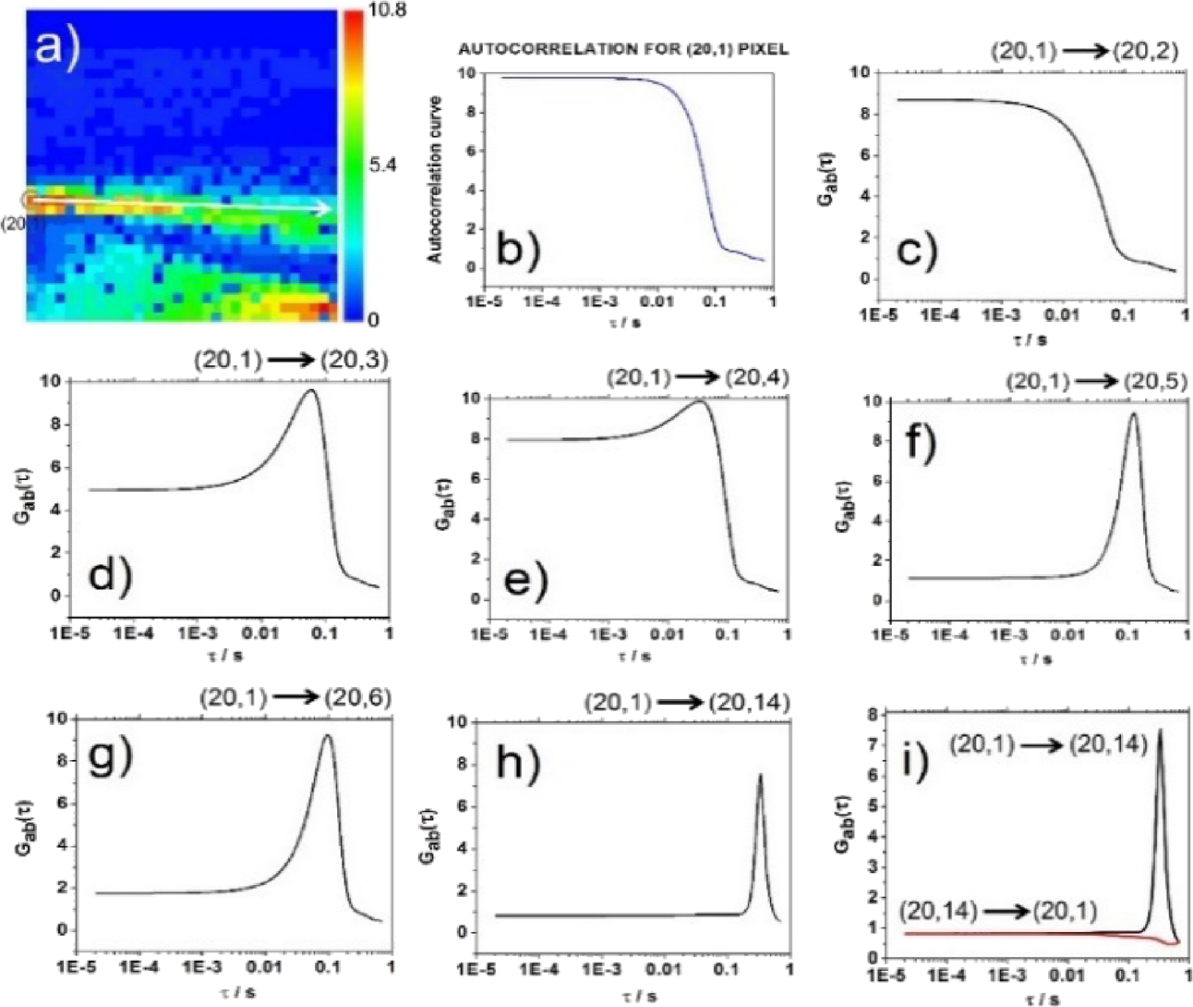
Two-foci cross-correlation analysis of directional particle motion in a diluted suspension of 2 μm fluospheres. (a) *G*(0) map showing amplitudes of temporal ACCs at lag time τ = 20.74 μs in the 32 × 32 SPAD array. The upper trail, starting at the referent SPAD *a* = (20,1), is encircled. The white arrow indicates the path along which two-foci cross-correlation analysis is performed. (b) The two-foci ACC at the referent SPAD *a* = (20,1) was calculated using eq 2 when *a* = *b*. (c–h) Two-foci cross-correlation curves (tfCCCs) between the referent pixel *a* = (20,1) and pixels *b* = (20,2) (c), *b* = (20,3) (d), *b* = (20,4) (e), *b* = (20,5) (f), *b* = (20,6) (g), and *b* = (20,14) (h). (i) tfCCC_ab_ between *a* = (20,1) and *b* = (20,14) (black; shown in panel h) and the corresponding tfCCC_ba_ between *b* = (20,14) and *a* = (20,1) (red). The absence of a peak in tfCCC_ba_ clearly indicates that the direction of particle motion is from (20,1) toward (20,14) and not from (20,14) toward (20,1).

**Figure 2:**
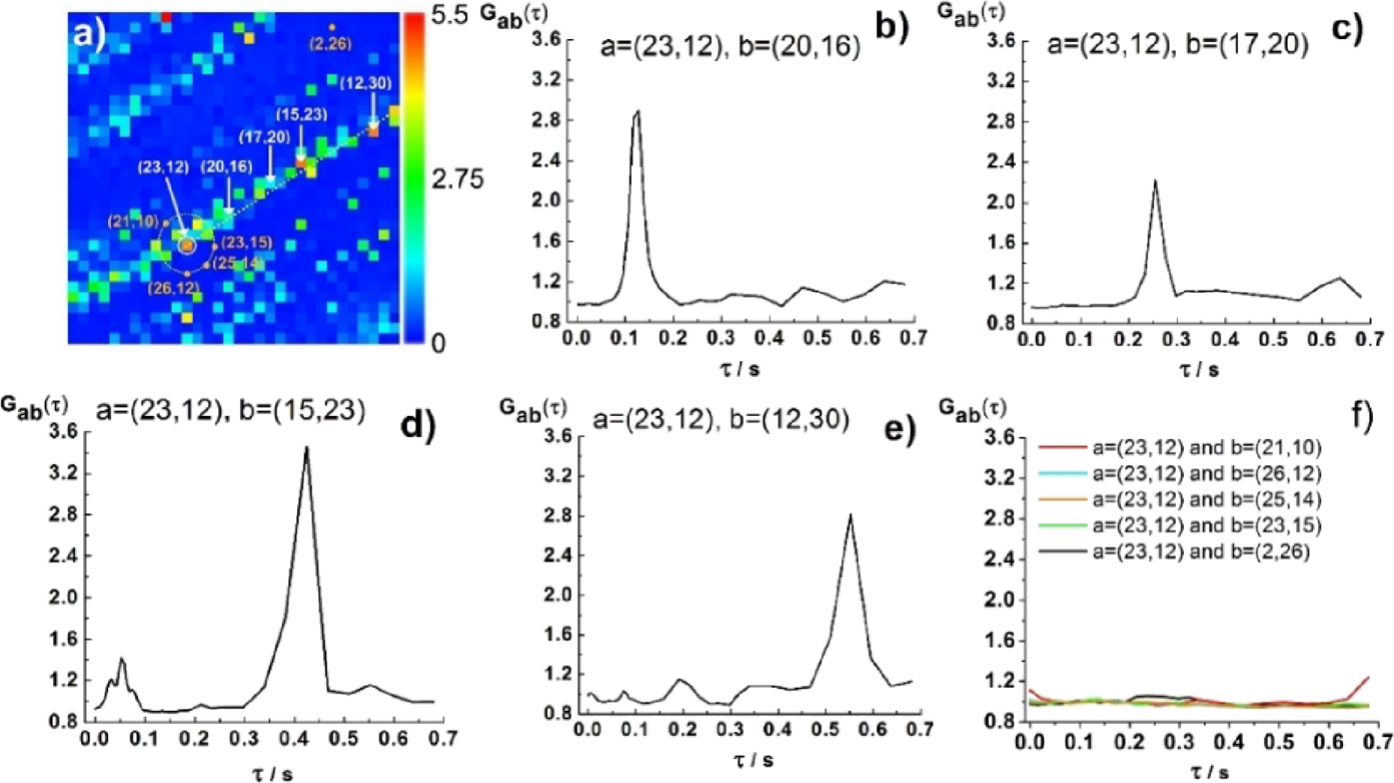
Two-foci cross-correlation analysis of directional particle motion in a 20 nM suspension of 100 nm fluospheres. (a) *G*(0) map showing amplitudes of temporal ACCs at lag time τ = 20.74 μs in the 32 × 32 SPAD array. To guide the eye, the trail of fluosphere motion, including the referent SPAD *a* = (23,12), is highlighted (white dotted line). (b–e) tfCCCs between the referent SPAD *a* = (23,12) and on-trajectory SPADs (white) *b* = (20,16), *b* = (17,20), *b* = (15,23), or *b* = (12,30). (f) tfCCCs between the referent SPAD *a* = (23,12) and selected off-trajectory SPADs (orange-marked).

Here, we demonstrate using dilute suspensions of fluospheres of different diameters, *d* = 2 μm (Figure 1) and *d* = 100 nm (Figure 2), that the projection of particles’ motion in the focal plane can be traced using two-foci cross-correlation analysis in mpFCS.

To begin with, a dilute suspension of large, *d* = 2 μm, and bright fluospheres is analyzed, where particle motion could readily be observed by temporal autocorrelation analysis of fluorescence intensity fluctuations acquired using the mpFCS system (Figure 1). As can be seen from the map of amplitudes of the temporal ACCs for lag time τ = 20.74 μs, the so-called *G*(0) map, high *G*(0) values are observed along paths reflecting fluosphere movement (Figure 1a). To ascertain that these traces indeed reflect particle motion, two-foci cross-correlation analysis is performed between selected pixels along the upper trail (Figure 1a, white arrow), and the thus-obtained two-foci cross-correlation curves (tfCCCs) are shown in Figure 1b–i.

The ACC in pixel (20,1) shown in Figure 1b is calculated for the referent SPAD *a* = (20,1) using eq 2 when *a* = *b*. The tfCCCs between two adjacent pixels *a* = (20,1) and *b* = (20,2) resemble the ACC shown in Figure 1b and lack the characteristic narrow peak that is characteristic of tfCCCs shown in Figure 1c–h. This is because of the large bead diameter, which exceeds the distance between the neighboring SPADs in the focal plane; the distance between the centers of two adjacent volume elements is Δ*l* = 1.6 μm. Thus, fluorescence signals from the same particle are simultaneously measured in both SPADs. tfCCCs containing features of both the ACC and tfCCC features are observed for *b* = (20,3) and *b* = (20,4) (Figure 1d,e). When the distance between two pixels is sufficiently large (Figure 1f–h), a sharp peak is observed, as expected. Importantly, one can see that the greater the distance between *a* and *b*, the longer the lag time of the cross-correlation peak. From the lag times of the cross-correlation peak, one can estimate the two-dimensional (2D) velocity. Projection of the three-dimensional (3D) fluosphere trajectory as it traverses the focal plane onto the 2D image plane divided by the lag time yields the 2D velocity. The 2D velocity at which the fluorescent particle is moving from pixel *a* = (20,1) is first 50 μm/s, then increases to 80 μm/s, and then decreases to 60 μm/s. Given that we have neither controlled the direction nor the velocity of fluosphere motion but rather observed its movement under spontaneous, convection or pipet aspiration/dispensing-driven fluid flow, the observed difference could reflect local changes in fluosphere velocity due to heterogeneities in the fluid flow and/or apparent differences in fluosphere 2D velocity arising because 3D paths of different lengths may give the same 2D projection in the image plane. Finally, the direction of fluosphere motion in the focal plane could be determined (denoted by the white arrow in Figure 1a) from the results shown in Figure 2i. Here, a sharp peak is observed when the tfCCC is calculated in the direction from *a* = (20,1) to *b* = (20,14) (Figure 2i, black). However, when the calculation is performed in the opposite direction, from *b* = (20,14) to *a* = (20,1), the peak disappears (Figure 2i, red). This in turn means that the direction of fluosphere motion is from *a* = (20,1) to *b* = (20,14).

Sensitivity of the mpFCS instrument is further tested using a diluted suspension of small fluospheres, *d* = 100 nm (Figure 2).

As can be seen from the *G*(0) map (Figure 2a) and the tfCCCs between the reference point *a* = (23,12) and pixels *b* = (20,16), *b* = (17,20), *b* = (15,23), or *b* = (12,30), robust peaks can be observed in the tfCCCs, confirming that these points are on the trajectory and in the direction of 100 nm fluosphere movement (Figure 2b–e). The greater the distance between pixels *a* and *b*, the longer the lag time at which the peak in the tfCCC is observed (Figure 2b–e). As before (Figure 1i), tfCCCs calculated from *b* to *a* (data not shown) showed no peaks, confirming that the direction of 100 nm fluosphere motion is from point *a* = (23,12) toward the set of white-marked *b* points along the indicated trajectory (Figure 2a, white dotted line). Moreover, from the lag times of the peaks in the tfCCCs (Figure 2b–e), the projection of mean velocity in the focal plane could be estimated, yielding an approximate mean velocity of the directed motion of 30 μm/s between pixels (23,12) and (22,13) and 60 μm/s thereafter. We next prove that two-foci cross-correlation analysis can be used as a “radar” for detecting the direction of particle motion. In Figure 2a, we depict a circle centered at the reference pixel *a* = (23,12) with pixels (21,10), (26,12), (25,14), and (23,15) at its rim (Figure 2a, orange-marked). In addition, the distant pixel (2,26) is also included in this analysis as an off-trajectory example (Figure 2a, orange-marked). tfCCCs between *a* = (23,12) and these pixels, which are not positioned along the particle trajectory, clearly show no peaks (Figure 2f). Thus, this 360° sweeping procedure can discern the direction of the particle motion. To further corroborate this analysis, we show in the Supporting Information (Section SI4, Figures S8–S10) that two-foci cross-correlation analysis yields no tfCCCs when pure diffusion is observed (Figures S8 and S9) and that it is more powerful than classical temporal autocorrelation analysis for discerning free diffusion from directional motion when the flow is slow (Figure S10).

### Nucleocytoplasmic Translocation of Glucocorticoid Receptor in the Live Cell

**Figure 3:**
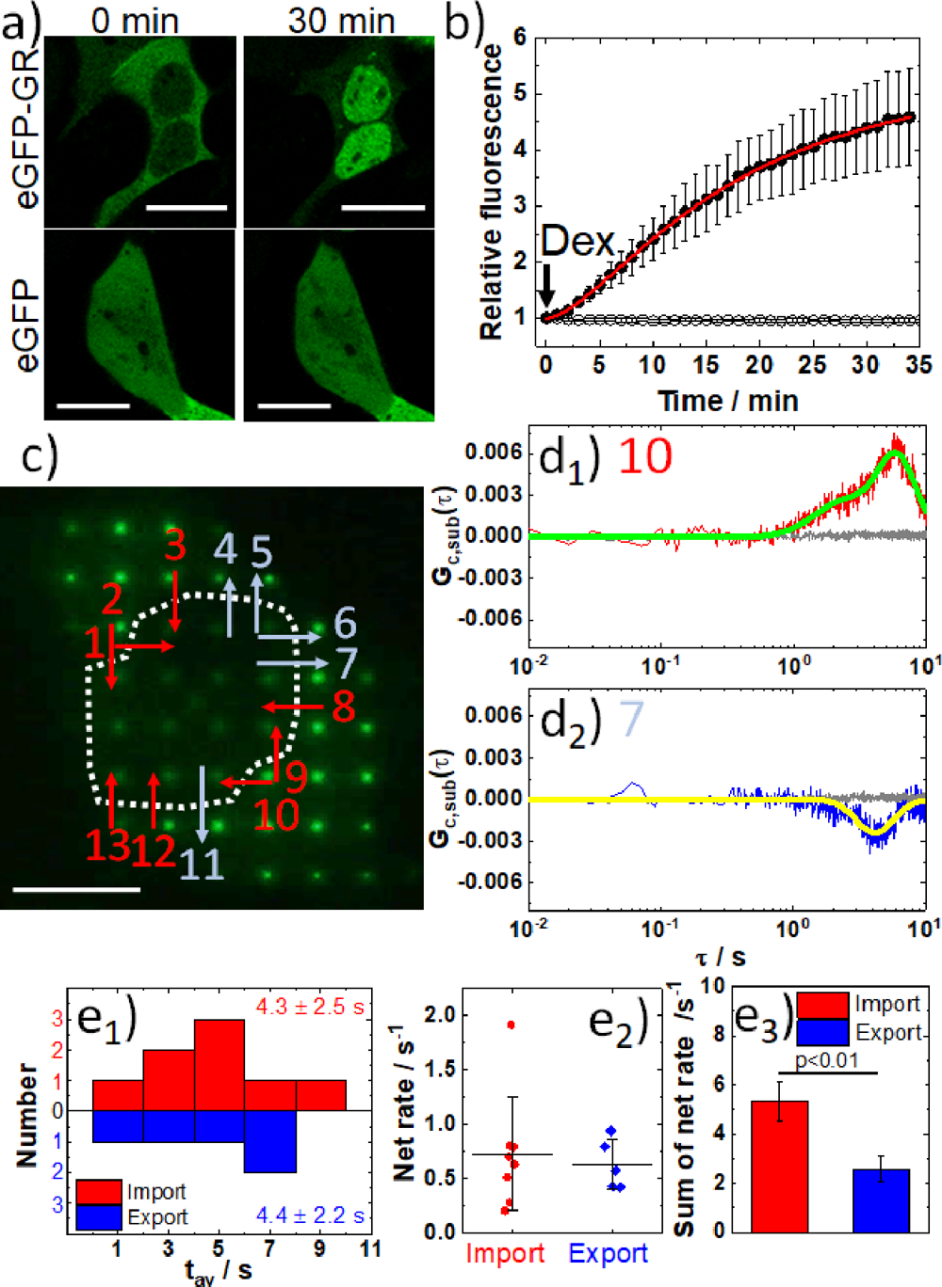
Spatial mapping of the direction and extent of nucleocytoplasmic transport of eGFP-GR in live cells using two-foci cross-correlation in mpFCS. (a) CLSM images of HEK cells expressing eGFP-GR (top) or eGFP (bottom; control) at 0 min (untreated) and 30 min after treatment with 500 nM Dex. Scale bars: 20 μm. (b) Relative change in nuclear fluorescence intensity over time following Dex treatment in cells expressing eGFP-GR (dots) or eGFP (circles). The arrow indicates the time of Dex addition to a concentration of 500 nM. Logistic function was used to fit the S-shaped relative fluorescence growth curve recorded in cells expressing eGFP-GR (red line). (c) Fluorescence image showing eGFP-GR distribution in an untreated cell acquired by using the DSRL Canon camera coupled to the side port of the mpFCS system. Arrows show the directions of eGFP-GR; nuclear translocation: import (red) and export (blue). Scale bar: 10 μm. (d_1_, d_2_) *G*_*c*,sub_(τ) at an import-active (red, d_1_) and export-active site (blue, d_2_). Translocation time is determined by Gaussian distribution fitting to *G*_*c*,sub_(τ) (green/yellow). (e_1_) Histogram of import (red) and export (blue) translocation time. (e_2_) Net rate of nucleocytoplasmic transport calculated as the ratio of the tfCCC amplitude over import/export time. (e_3_) Sum net rate in a single cell. All values are shown as the average ± standard deviation (SD). SD was calculated from three independent measurements in three cells. Statistical significance was assessed using a two-tail Student’s *t* test.

Finally, we applied our method to characterize in live cells the translocation of the glucocorticoid receptor tagged with enhanced green fluorescent protein (eGPFP-GR; Figure 3).

As can be seen, eGFP-GR is localized in the cytoplasm in untreated HEK cells (Figure 3a, top, 0 min). Dex (500 nM) treatment induced eGFP-GR relocation to the cell nucleus (Figure 3a, top, 30 min), whereas it did not change eGFP intracellular distribution (Figure 3a, bottom, 0 and 30 min). Over time, a 4-fold increase in nuclear fluorescence intensity following 500 nM Dex treatment was observed in eGFP-GR-expressing HEK cells (Figure 3b, dots), while no change was observed in the eGFP-expressing HEK cells (Figure 3b, circles). eGFP-GR nuclear translocation half time, defined as the time needed for fluorescence intensity in the cell nucleus to reach half of its highest value, was determined to be *t*_1/2_^nuc^ = 15 min (Figure 3b, red). Hence, mpFCS measurements of directional transport were performed in eGFP-GR-expressing HEK cells 15 min after the treatment with 500 nM Dex.

Nuclear/cytoplasmic positions of eGFP-GR in an untreated HEK cell expressing eGFP-GR are visualized using spot-wise illumination and the DSLR camera attached to the side-port of the mpFCS system (Figure 3c; nuclear localization is inside the white dotted contour). tfCCCs, *G*_*c*_^cyt,nuc^(τ) and *G*_*c*_^nuc,cyt^(τ), and corresponding *G*_*c*,sub_(τ) are calculated at distinct positions along the nuclear envelope (Figure 3c, 1–13). Our data showed that the direction of nuclear import/export is location-specific, with nuclear import being observed at sites 1, 2, 3, 8, 9, 10, 12, and 13 (Figure 3c, red), whereas nuclear export was observed at sites 4, 5, 6, 7, and 11 (Figure 3c, faint blue).

Representative *G*_*c*,sub_(τ), color-coded to indicate the direction of eGFP-GR nucleocytoplasmic transport, nuclear import (red) and nuclear export (blue), are shown in Figure 3d_1_,d_2_, respectively. *G*_*c*,sub_(τ) for all 13 sites are shown in the Supporting Information (Section SI5, Figure S11a). Average *G*_*c*,sub_(τ) for three different cells, showing similar results, are presented in Figure S11b_1_–b_3_.

We further computed the nucleocytoplasmic translocation times (Figure 3e_1_) by fitting the *G*_*c*,sub_(τ) by using a Gaussian distribution function (Figure 3d_1_,d_2_, green and yellow, respectively). On the average, the nucleocytoplasmic translocation times were *t*_av_^imp^ = (4.3 ± 2.5) s for nuclear import and *t*_av_^exp^ = (4.4 ± 2.2) s for nuclear export (Figure 3e_1_). By dividing the amplitude of the fitted Gaussian distribution function with the corresponding nucleocytoplasmic translocation time, the net rate of nucleocytoplasmic translocation could be estimated at independent sites (Figure 3e_2_) and summed at all analyzed sites (Figure 3e_3_).

This analysis, corroborated by theoretical calculations (Section SI5, Figures S12–S14), showed that on average, net rate of nucleocytoplasmic translocation at different sites along the nuclear envelope was not significantly different between nuclear import and export sites (Figure 3e_2_). However, given that the number of sites at which nuclear import was observed is larger than the number of sites where nuclear export occurred, the total import net rate was twice the total export net rate (Figure 3e_3_), consistent with the net eGFP-GR translocating into the cell nucleus.

## Discussion

In this work, we have theoretically and experimentally shown that two-foci cross-correlation analysis in mpFCS enables us to characterize the directional motion of fluospheres/molecules in solution/live cells. This is exemplified here for fluospheres of different sizes, *d* = 2 μm, which is larger than the distance between two adjacent pixels in the mpFCS instrument (Figure S1), and *d* = 100 nm, which is smaller, in dilute aqueous suspension (Figures 1 and 2 and Figures S2–S5), for immobilized q-dots precisely moved using a translation stage with nanometer step size (Figures S6 and S7), and for ligand-induced eGFP-GR nuclear translocation in live cells (Figure 3 and Figure S11).

Using dilute suspension of fluorescent particles, we demonstrated theoretically and experimentally that by calculating tfCCCs between a referent pixel and different adjacent pixels in the focal plane, we could identify the direction in which the particle is moving since a sharp peak is observed only in the tfCCC that is calculated along the projection of the 3D path of particle motion onto the 2D image plane as it traverses the focal plane and in the direction of particle movement from an earlier to a later particle position (Figures 1 and 2). Moreover, we showed that the tfCCC peak is observed at the lag time that corresponds to the transit time between the two foci positions, demonstrating that our technique can be used not only to trace the projection of the 3D particle trajectory onto the 2D image plane as the particle traverses the focal plane but also to map the forward/backward direction of its motion and the 2D velocity.

We further showed using ligand-induced eGFP-GR nuclear translocation as an example that two-foci cross-correlation analysis can characterize in live cells the cellular dynamics of fluorescently tagged proteins. Most notably, our study showed that by calculating tfCCCs, we could map the direction of ligand-induced eGFP-GR nucleocytoplasmic translocation along the nuclear envelope contour in the focal plane (Figure 3). This further revealed that eGFP-GR nuclear import and export occurred simultaneously at different locations along the nuclear envelope and with similar nucleocytoplasmic translocation times, *t*_av_^imp^ = (4.3 ± 2.5) s for nuclear import and *t*_av_^exp^ = (4.4 ± 2.2) s for nuclear export (Figure 3e_1_), and that the net effect at the macroscopic level is determined by the number of sites through which import/export occurs (Figure 3e_1_–e_3_). We thus confirmed a previous finding by multipoint holographic fluorescence correlation spectroscopy (MP-hFCS)^22^ showing that under ligand-induced eGFP-GR nuclear translocation, the actual passing through of nuclear pores is faster than the macroscopic change in fluorescence intensity that is observed by time-lapse confocal fluorescence microscopy imaging. We, however, took the understanding of mechanisms underlying acute, strong-binding ligand-induced eGFP-GR translocation one step further by showing that import/export processes occur simultaneously at different locations, which was not shown by MP-hFCS, and that the net change in eGFP-GR concentration at the macroscopic level is determined by the number of sites through which eGFP-GR import/export occurs and not by differences in the actual eGFP-GR translocation time (Figure 3e_1_–e_3_). Having said this, we underline here that this conclusion is valid only for acute, strong-binding ligand-induced eGFP-GR nuclear translocation at the time of the highest net change in eGFP-GR nuclear concentration and does not necessarily apply for other conditions, such as under stimulation with endogenous or synthetic weakly binding GR-specific ligands or upon withdrawal of GR ligand.^51−54^

In conclusion, two-foci cross-correlation in mpFCS is a versatile tool for characterization of both direction and 2D velocity of active transport in live cells and may be useful for understanding how the bidirectional traffic of macromolecules is spatially organized and coordinated in live cells to achieve controlled local accretion/abatement of molecules of interest. In addition, directional fluorescent particle motion in fluids can be characterized in detail, enabling us not only to characterize local heterogeneities in fluid flow but also to investigate the essential role of the fluid phase for long-range material transport and the emergence of large-scale synergy in biological systems.^55^

## Supporting Information Available

SI1: optical setup; SI2: theoretical background and numerical simulations of directional particle motion; SI3: proof-of-concept measurements using a piezo-based linear stage with nanometer positioning precision for controlled translation of immobilized quantum dots; SI4: comparison between auto- and two-foci cross-correlation analysis of diffusion and flow in dilute aqueous solution and nanoparticles in suspension; SI5: glucocorticoid receptor nucleocytoplasmic translocation in live cells (PDF)
