# Supplementary material for: Mapping the Direction of Nucleocytoplasmic Transport of Glucocorticoid Receptor (GR) in Live Cells Using Two-Foci Cross-Correlation in Massively Parallel Fluorescence Correlation Spectroscopy (mpFCS)
Source: Anal Chem. 2023 Oct 2;95(41):15171–9. doi: 10.1021/acs.analchem.3c01427 (PMC10585663; doi:10.1021/acs.analchem.3c01427)
Supplement: Supplementary file 1 — ac3c01427_si_001.pdf [file ac3c01427_si_001.pdf]

Supporting Information: Mapping the direction of nucleocytoplasmic transport of glucocorticoid receptor (GR) in live cells using two-foci cross-correlation in massively parallel Fluorescence Correlation Spectroscopy (mpFCS)

Stanko N. Nikolić[1,2,3,¶], Sho Oasa[1,¶], Aleksandar J. Krmpot[1,2,3,¶], Lars Terenius[1], Milivoj R. Belić[3], Rudolf Rigler[1,‡,†], Vladana Vukojević[1, ‡,*]

[1]Department of Clinical Neuroscience (CNS), Center for Molecular Medicine (CMM), Karolinska Institute, 17176 Stockholm, Sweden. [2]Institute of Physics Belgrade, University of Belgrade, 11080 Belgrade, Serbia. [3]Division of Arts and Sciences, Texas A&M University at Qatar, Doha, Qatar.

ABSTRACT: SI1: Optical setup. **SI2:** Theoretical background and numerical simulations of directional particle motion. **SI3:** Proof of concept measurements using a piezo-based linear stage with nanometre positioning precision for controlled translation of immobilized quantum dots. **SI4:** Comparison between auto- and two-foci cross-correlation analysis of diffusion and flow in dilute aqueous solution and nanoparticles in suspension. **SI5:** Glucocorticoid receptor nucleocytoplasmic translocation in live cells.

SI1: Optical setup.
Schematic drawing of the optical setup of the mpFCS system is shown in Fig. S1.

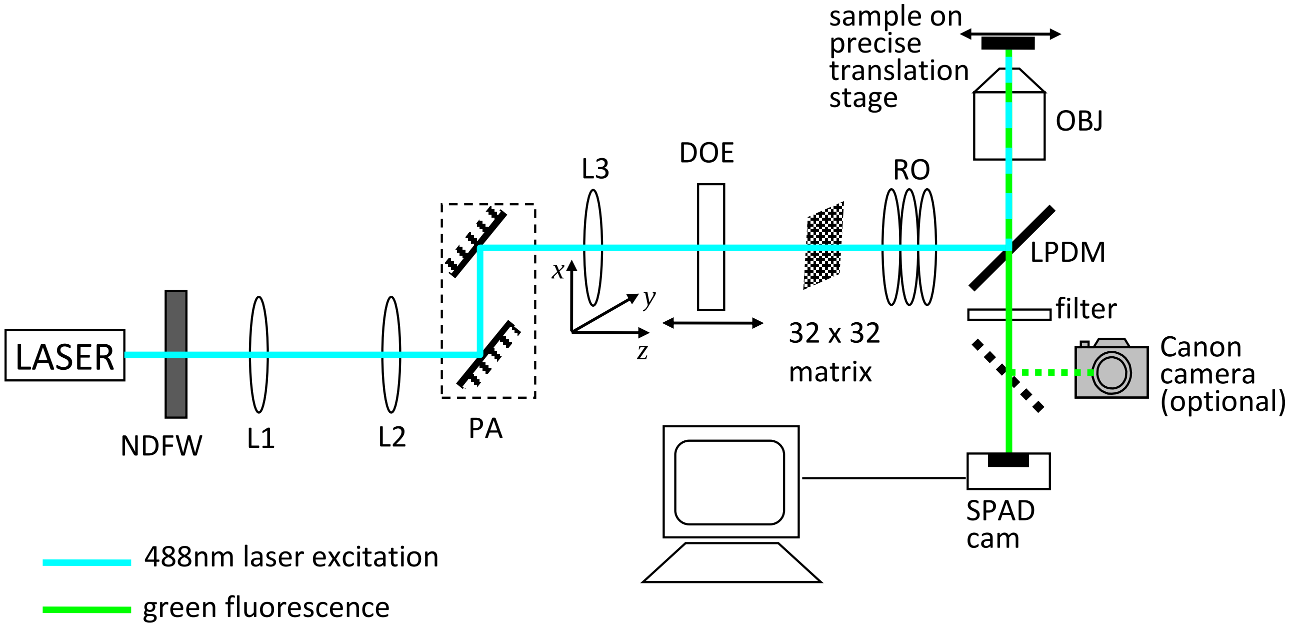

Figure S1. Optical setup for mpFCS. NDFW - neutral density filter wheel, L1 and L2 – beam expander lenses, PA – periscope assembly, L3 – focusing lens, DOE – diffractive optical element, RO – relay optics of the microscope, LPDM – long pass dichroic mirror, OBJ – microscope objective, SPAD cam – single photon avalanche photodiode camera. The laser beam passes through a double filter wheel of different optical density (OD 0.2-8.0) that can be adjusted in discrete steps for precise regulation of illumination intensity. Uniform filters are used rather than a variable one in order to avoid wave front distortion and thus preserve symmetrical light intensity distribution in the focus. The laser beam is than expanded about 10 times by a Keplerian telescope (L1 and L2). The expanded beam is focused by the plano-convex lens L3 through the diffractive optical element (DOE) designed to split a single laser beam into 32×32 beams. A 32×32 foci illumination matrix is formed at the rear port

image plane of the epi-fluorescence microscope Axio Observer D1. The relay optics of the rear port, dichroic mirror, and the C-Apochromat 63x/1.2 W Corr Objective transfer the illumination matrix image into the focal plane of the objective. Translation of L3 in x and y direction scans the incidence angle of the laser beam to the rear port of the microscope and tunes the illumination matrix lateral position in the object plane. Translation of L3 in z direction adjusts sureness of the matrix. The DOE can be translated along the beam propagation direction enabling the dimension *i.e.* the pitch of the illumination matrix to be adjusted in order to match the detector's matrix. The Zeiss Filter Set 38 HE for enhanced Green Fluorescent Protein (eGFP) consisting of an excitation band pass filter EX BP 470/40 nm (central wavelength/band width), long pass dichroic mirror with a cut-off wavelength of 495 nm, and an emission band pass filter EM BP 525/50 was used throughout. Fluorescence was detected using the SPC2 camera, containing a photosensitive chip and a 16-bit photon counter based on Field Programmable Gate Array (FPGA). The photosensitive area of the chip consists of 32×32 circular SPADs that are 20 μm in diameter. The distance between adjacent diodes in the same row/column is 100 μm. Further details on the SPC2 camera design and performance can be found in [36, 41-43]. Since the aperture of every SPAD in the SPC2 camera acts as a pinhole positioned in a conjugate focal plane with respect to the illumination matrix, confocal configuration is achieved for all 32×32 foci.

SI2: Theoretical background and numerical simulations of directional particle motion.

SI2a: Theoretical model.

To calculate the photon counts when a single particle (such as a fluosphere or a quantum dot) is travelling in the focal plane of the experimental setup, the particle motion is assumed to be two-dimensional. The frame duration in the model is taken to be the same as in the experiment: dt = 20.74 μs. For simplicity, the origin of the reference frame is placed at the centre of the first observation volume element (1,1). If we denote the distance between the centres of two adjacent volume elements with $\Delta l = 1.6$ μm, then the coordinates of the centre of element with indices (i,j) are: $y_{i,j} = (i-1)\Delta l$ and $x_{i,j} = (j-1)\Delta l$ $(1 \leq i,j \leq 32)$.

The laser radial intensity distribution of each volume element is Gaussian:

$$I = I_0 e^{-8r^2/D^2} \tag{S1}$$

where r corresponds to radial distance of an arbitrary point in the focal plane from the centre of the volume element *(i,j)* and D is a diameter of the beam (1/e^2 definition). Let the particle take initial position $\boldsymbol{r_{0p}}$ and velocity $\boldsymbol{v}$, while its radius is $r_p = d/2$. Then, its coordinates at a given moment t are:

$$x_p = (\boldsymbol{r_{0p}} + \boldsymbol{v}t)\boldsymbol{e_x} \tag{S2}$$

$$y_p = (\boldsymbol{r_{0p}} + \boldsymbol{v}t)\boldsymbol{e_y} \tag{S3}$$

The number of photons $dN_{\text{photons}}(i,j,t)$ measured in the camera pixel corresponding to a volume element (i,j) during infinitesimally time interval (t, $t + dt$) is calculated by using the following equation:

$$dN_{photons}(i,j,t) = \alpha dt \oiint I(r)\eta'(r,\varphi,x_p,y_p)d^2S \tag{S4}$$

Our assumption is that the number of photons is proportional to the: 1. incident laser intensity at particular point of the volume element with polar coordinates r and φ (in the second referential frame with origin in the center of (i, j) element), 2. small area $d^2S = rdrd\varphi$ around that point, and 3. time interval dt. This applies if surface d^2S fully/partially overlaps with the particle. In addition, in the focal plane, the particle is considered as a circle of radius $r_p^2\pi$. Therefore, $\eta'(r,\varphi,x_p,y_p)$ indicates "overlapping" function: it is equal to one if a given point of a volume element (i,j) with coordinates (r, φ) is "inside" the particle at time t. Otherwise, $\eta'(r,\varphi,x_p,y_p)$ is zero. If we use η function defined as:

$$\eta(x) = 1 \ (x \geq 0)$$
$$\eta(x) = 0 \ (x < 0) \tag{S5}$$

we can write:

$$\eta'(r,\varphi,x_p,y_p) = \eta\left[r_p^2 - \left((j-1)\Delta l + rcos\varphi - x_p\right)^2 - \left((i-1)\Delta l + rsin\varphi - y_p\right)^2\right] \tag{S6}$$

Finally, the eq. (S6) can be written as:

$$dN_{\text{photons}}(i,j,t) = \alpha I_0 \int_{r=0}^{\beta D/2} rdr \cdot \int_{\varphi=0}^{2\pi} d\varphi \cdot e^{\frac{-8r^2}{D^2}} \cdot \eta\left[r_p^2 - \left((j-1)\Delta l + rcos\varphi - x_p\right)^2 - \left((i-1)\Delta l + rsin\varphi - y_p\right)^2\right] \tag{S7}$$

In last equation, α is proportionality constant. We take additional parameter β = 1.1 to define the region of integration around the center of the volume element (i,j).

SI2b. Particle motion along the x-axis.

Setting overall simulation time (T), time step (Δt), and number of frames (N_{total}) to their experimental values: T = 2.71 s, Δt = 20.74 us, and N_{total} = 2.71 s/20.74 μs ~ 131000, we compute the photon counts $n_a(t)$ and $n_b(t)$ at two chosen pixels a and b, respectively, using eq. (S7). Then we calculate tfCCCs by placing $n_a(t)$ and $n_b(t)$ into eq. (2).

Here we provide results of numerical simulations when fluorescent particle is moving along the x-axis: $x_{0p} = y_{0p} = 0, \boldsymbol{v} = 40$ μm/s $\boldsymbol{e_x}, D = 560$ nm, $\Delta l = 1.6$ μm . We varied the size of the particle: d = 100 nm or d = 2 μm.

For a small particle with d = 100 nm, the calculated tfCCCs are shown in Fig. S2a-c. The volume element (1,1) is

fixed as pixel a. The partner pixel b for tfCCC calculation was varied as (1,2), (1,3) and (1,5), respectively.

The lag times of peaks in tfCCCs correspond to the travelling time of the 100 nm particle between two pixels. The average flight times are 0.04 s (for Δl), 0.08 s (for $2\cdot\Delta l$), and 0.16 s (for $4\cdot\Delta l$). The projection of the particle velocity in the focal plane is determined by the ratio of the distance between a and b to the position of the peak. From Fig. S2, $v = 1\cdot\frac{\Delta l}{t_1} = 2\cdot\frac{\Delta l}{t_2} = 4\cdot\frac{\Delta l}{t_3} \sim 40$ μm/s. This is a theoretical proof that a particle velocity in the focal plane can be computed from tfCCC.

Next, we conducted numerical simulation when particle size (d = 2 μm) is larger than confocal volume element and pixel-to-pixel distance Δl (Fig. S2d-f). The tfCCCs computed in this case are also characterized by peaks appearing at particular time instants: 0.043 s, 0.083 s, and 0.17 s for Figs S2d-f, respectively. In contrast to a 100 nm fluosphere (Fig. S2a), the tfCCC in Fig. S2d is composed of a wider peak standing on a high background before the peak has appeared. This high background is decreased (Fig. S2e), finally dropping to zero in Fig. S2f. When the particle radius is smaller, the photons are emitted from a single volume element at a time. This means that photon counts at different camera pixels are correlated only when time lag is equal to the time of flight. On the other hands, when diameter is large enough ($2r_p \geq \Delta l - D$), then particle can emit photons from different volume elements at the same time. The correlation then appears for time lags shorter than time of flight, which results in high cross-correlation values before the characteristic peak. In addition, the tfCCC peak is wider for larger particles (since it needs more time to pass through the entire volumes of two foci), making the velocity determination method from peak position less accurate.

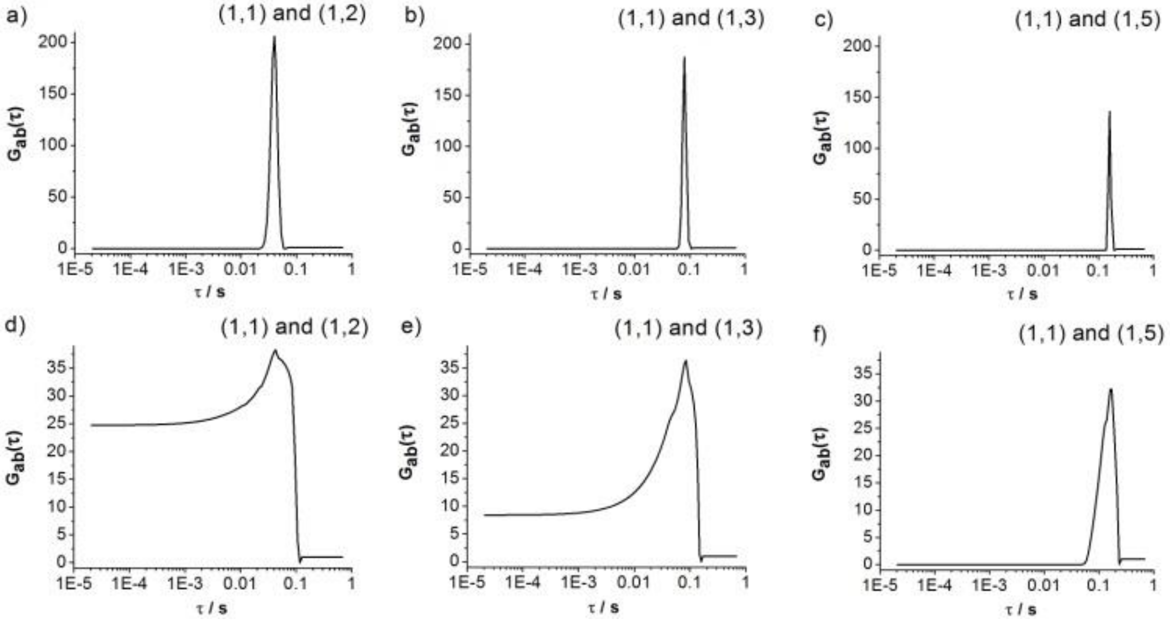

Figure S2. Theoretical simulation of particle movement. The two-foci cross-correlation curves (tfCCCs) corresponding to different pairs of volume elements that the 100 nm fluosphere **(a-c)** and 2 μm fluosphere **(d-f)** is passing through. The pixel pairs (a,b) are: a,d) (1,1) and (1,2), b,e) (1,1) and (1,3), c,f) (1,1) and (1,5). The particular peak in each graph indicates the time that a particle needs to arrive from pixel a and pixel b. **(a-c)** The peak positions on time axis are (a) t_1 = 0.04 s, (b) t_2 = 0.08 s, and (c) t_3 = 0.16 s for each pixel-pixel distances Δl, $2\cdot\Delta l$, and $4\cdot\Delta l$. Calculated velocity is 40 μm/s. **(d-f)** The peak positions on time axis are (d) t_1 = 0.043 s, (b) t_2 = 0.083 s, and (c) t_3 = 0.17 s for each pixel-pixel distances Δl, $2\cdot\Delta l$, and $4\cdot\Delta l$. Calculated velocity is 40 μm/s.

SI2c. The trajectory of particle motion at a non-zero angle with respect to the *x*-axis.

When the projection of particle velocity in the focal plane is oriented at a non-zero angle with respect to *x*-axis, then the speed can be determined from the tfCCC calculated between pixels that lie on a line which intercepts the *x*-axis under the same angle. To find the pixels pair for tfCCCs computation, the autocorrelation curves were determined first (Fig. S3).

For a d = 100 nm particle, single trace is obtained on the autocorrelation curves amplitude map. When 2 μm particle passes across the camera, its fluorescence signal is simultaneously detected in adjacent camera pixels leading to a "smeared" trace. The traces of particles in case of α = 30° angle between velocity vector and *x*-axis are shown in Figs S3a (d = 100 nm) and S3b (d = 2 μm). The results for α = 45° are shown in Figs S3c (d = 100 nm) and S3d (d = 2 μm).

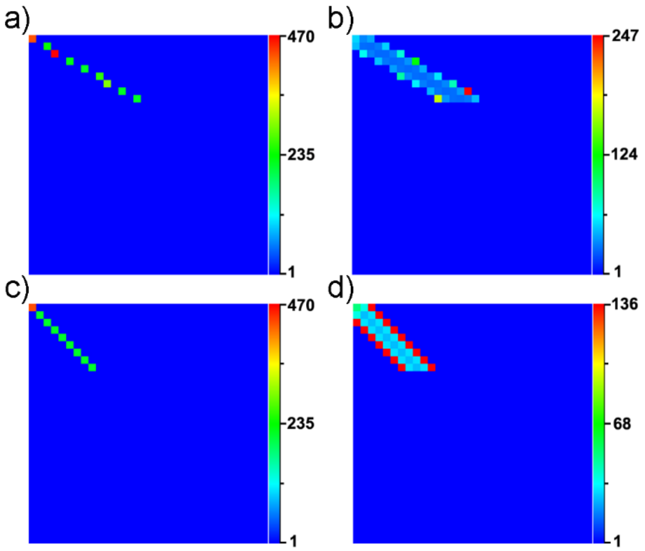

Figure S3. The amplitude of ACCs calculated up to the ninth row using theoretically obtained photons counts. Fluospheres diameter, *d*, and angle of motion (α) are: **(a)** d = 100 nm, α = 30°, **(b)** d = 2000 nm, α = 30°, **(c)** d = 100 nm, α = 45°, **(d)** d = 2000 nm, α = 45°.

The calculated tfCCCs for the angle α = 30° are shown in Fig. S4. Here, Figs S4a,b show data for a particle of diameter d = 100 nm and S4c,d for a particle d = 2 μm. The tfCCCs are calculated between the two volume elements (a,b) that a particle is passing through: a), c) (1,1) and (2,3), and b), d) (1,1) and (9,15). The positions of the peaks on the time axis are measured as: (a) 0.086 s, (b) 0.64 s, (c) 0.088 s, and (d) 0.61 s.

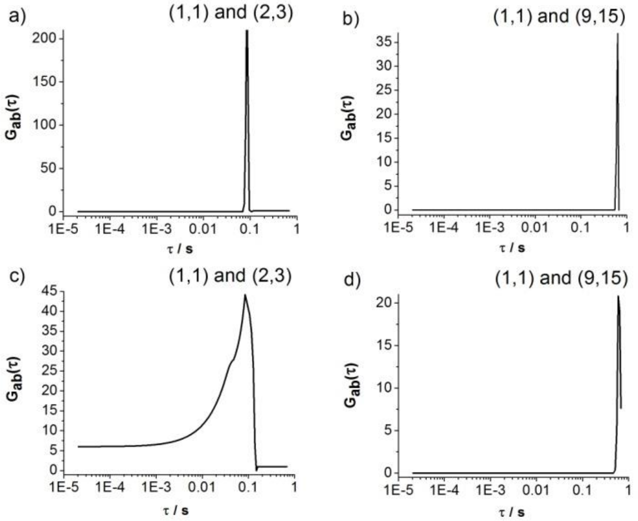

Figure S4. **tfCCCs between the two volume elements (*a*,*b*). (a)** (1,1) and (2,3), **(b)** (1,1) and (9,15), **(c)** (1,1) and (2,3), and **(d)** (1,1) and (9,15). The angle between velocity projection in the focal plane and x-axis is α = 30°. Graphs shown in a) and b) correspond to particle of diameter d = 100 nm, while graphs shown in (c) and (d) correspond to particle diameter of d = 2 μm.

The theoretically preset velocity is exactly 40 μm/s, while the calculated values are: (a) 41.4 μm/s, (b) 40.2 μm/s, (c) 40.5 μm/s, and (d) 41.8 μm/s. The relative errors between exact and calculated speeds are less than 5 % which makes this method appropriate for measuring the velocity component along any direction in the focal plane.

We also show the tfCCCs when the angle of motion is α = 45° (Fig. S5).

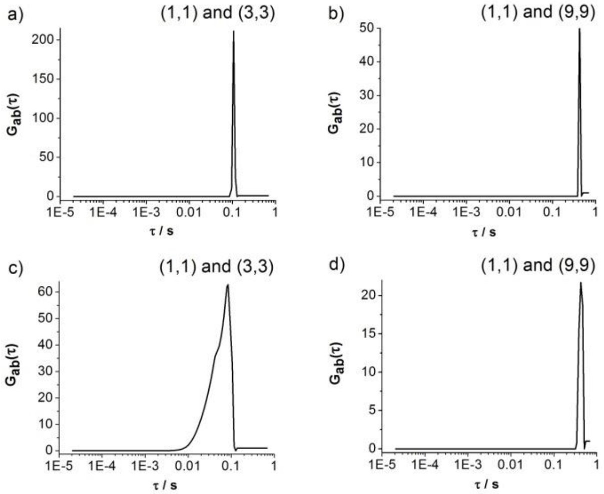

Figure S5. **tfCCCs between the two volume elements (*a*,*b*). (a)** (1,1) and (3,3), **(b)** (1,1) and (9,9), **(c)** (1,1) and (3,3), and **(d)** (1,1) and (9,9). The angle between velocity projection in the focal plane and x-axis is 45°. Figures (a) and (b) correspond to particle diameter of 100 nm, while figures (c) and (d) to particle diameter of 2 μm.

The tfCCCs were calculated between the two volume elements (a,b) that a particle is passing through: a), c) (1,1) and (3,3), and b), d) (1,1) and (9,9), for 100 nm and 2 μm particles. The peaks positions on time axis are measured as: (a) 0.11 s, (b) 0.42 s, (c) 0.084 s, and (d) 0.44 s. The exact velocity applied in a simulation is 40 μm/s, while the calculated values are: a) 41.9 μm/s, b) 42.8 μm/s, c) 53.5 μm/s, and d) 41.3 μm/s. The relative errors between exact and measured velocities are less than 5% for 100 nm particle (Figs S5a,b). However, the relative error was determined to 33% in case of 2 μm bead for a shorter pixel-to-pixel distance (Fig. S5c). The observed discrepancy could be explained in terms of very wide tfCCC maximum due to large particle diameter and a small pixel-to-pixel distance. This implicates that for certain angles and larger particles, the velocity should be calculated between the relatively distant pixels pairs.

S3: Proof-of-concept measurements using a piezo-based linear stage with nanometre positioning precision for controlled translation of immobilized quantum dots.

To validate the theoretical concepts under strictly controlled directional fluorescent particle motion, quantum dots (q-dots) immobilized on a glass surface were mounted

on a precise translation stage with a nanometer step size and the stage velocity was set to v_{stage} = 40 μm/s in the *y*-axis direction. The q-dot sample was translated over a specified distance and then rapidly, at a velocity v_{stage}^{return} >> 40 μm/s, brought back to the starting position. This procedure was repeated twice thereafter. Fluorescence intensity fluctuations collected using the SPC2 32×32 SPAD array camera were first analysed using temporal autocorrelation analysis (Fig. S6). Spatial mapping of the amplitudes of the auto-correlation curves (ACCs) at lag time τ = 20.74 μs, G(0), shows that the highest *G*(0) value was measured in pixel (7,29). The linear trajectory of the q-dot translation motion that started from pixel (7,29) is clearly visible across several rows in the 29th column (Fig. S6a). The actual ACC at pixel (7,29) is shown in Fig. S6b. The sharp peaks at long lag times reflect the moments when the same particle has entered pixel (7,29) for the second and the third time, respectively.

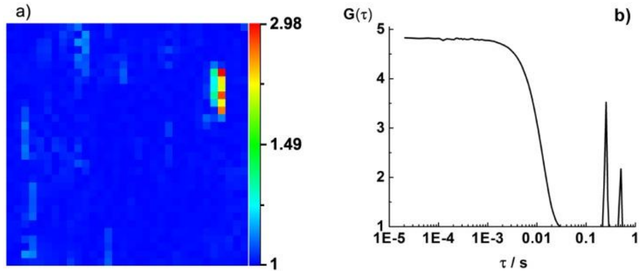

Figure S6. Autocorrelation measurements of immobilized q-dots translatory moved in the y-axis direction using a motorized stage. (a) The G(0) map showing amplitudes of temporal ACCs at lag time τ = 20.74 μs in the 32×32 SPAD array. **(b)** The ACC in pixel (7,29).

Of note, the characteristic decay time of the ACC in pixel (7,29), τ_c = 0.014 s, when multiplied with the pre-set velocity of the translational stage movement, v_{stage} = 40 μm/s, yields the diameter of the focal element: $d_F = v_{stage} \cdot \tau_c$ = 560 nm. This value is in a good agreement with the focal diameter ω_{xy} = (510 ± 90) nm previously determined in mpFCS calibration experiments.[1]

Next, we calculated the tfCCCs corresponding to the different pairs of pixels along the trajectory generated by immobilized q-dots movement by stage translation along the *y*-axis. The results for pixel pairs (*a*,*b*) are presented in Fig. S7: a) (7,29) and (8,29), b) (7,29) and (9,29), c) (7,29) and (11,29), and d) (7,29) and (13,29). The lag times at which peaks in each tfCCC are observed indicates the q-dot arrival time to pixel *b* from pixel *a.* The first peak in figure a) corresponds to the travel time τ_1 of a particle between the *i*) first, *ii*) second, and *iii*) third subsequent arrivals in *a* and *b*. The second peak is measured at time τ_2, representing two events: *i*) the first arrival in *a* and the second arrival in *b,* and *ii*) the second arrival in *a* and the third arrival in *b*. Similarly, the third peak denotes the time interval between the first arrival in *a* and the third arrival in *b*. Since fewer events are involved as time lag progresses, each subsequent peak has a lower amplitude. It should be noticed that all peaks are equidistant, *i.e.*, the time between adjacent peaks is Δτ = 0.255 s. The velocity of quantum dots is determined by the ratio of the distance between *a* and *b* to the position of the first peak. We have measured the velocity of q-dots in each graph a)-d), and the measured value agreed well with the value by which the translation stage was set to move.

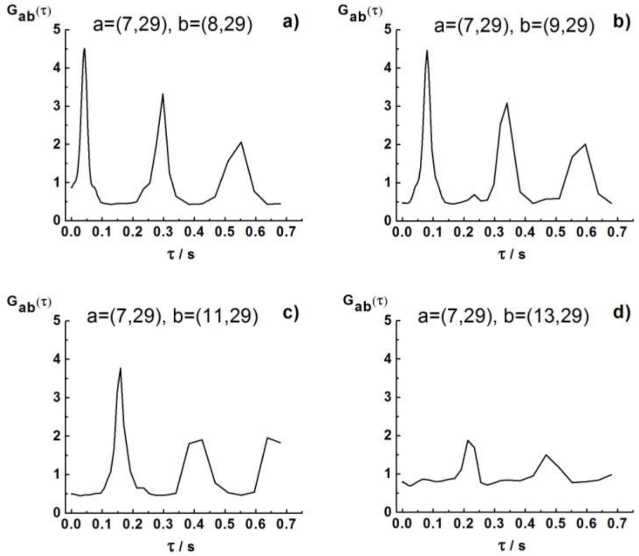

Figure S7. The tfCCCs between different pixels in case of translation motion of quantum dots. The pixel pairs (*a*,*b*) are: **(a)** (7,29) and (8,29), **(b)** (7,29) and (9,29), **(c)** (7,29) and (11,29), and **(d)** (7,29) and (13,29).

The velocity can be simply calculated from Fig. S7c): *v* = 4·1.59 μm / 0.158 s = 40 μm/s, as was adjusted at the translation stage. From Figs S7a to S7d, one can notice that if pixels *a* and *b* are at larger distance, the first peak is shifted on the time axis. This is expected since the particle will take longer time to reach *b* from *a*. Note that the time scales in all figures are linear, so the reader could easily observe equidistant peaks and thus the constant velocity of the q-dots, *i.e.* stage, movement.

SI4: Comparison between auto- and two-foci cross-correlation analysis of diffusion and flow in dilute aqueous solution and nanoparticles in suspension

To verify the procedure for two foci cross-correlation, we show using dilute solution of eGFP, 100 base pairs double-stranded dual-colour labelled DNA (dc-DNA) and fluospheres (diameter, d = 20 nm) that tfCCCs for pure diffusion is zero (Fig.S8).

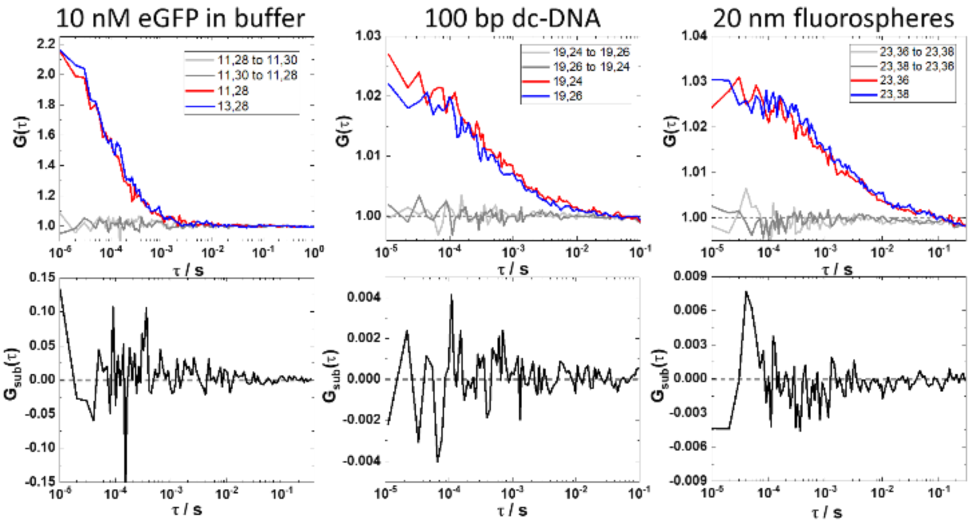

Figure S8. Spatial mapping of the direction and extent of transport in solution using two-foci cross-correlation in mpFCS. *Upper row:* Autocorrelation curves in individual pixels (red and blue) and corresponding cross-correlation curves calculated in opposite directions (light and dark grey) recorded in an aqueous buffer solution of eGFP (left), 100 bp ds-DNA (middle) and 20 nm fluospheres (right). *Lower row:* Corresponding $G_{c,sub}(\tau)$ obtained by subtracting the two-foci cross-correlation curve calculated in one direction, from the two-foci cross-correlation curve calculated in the opposite direction.

Moreover, we further corroborate the "360° sweeping procedure" by showing the results of two-foci cross-correlation analysis between all adjacent pixels for eGFP that undergoes free 3D diffusion only (Fig. S9).

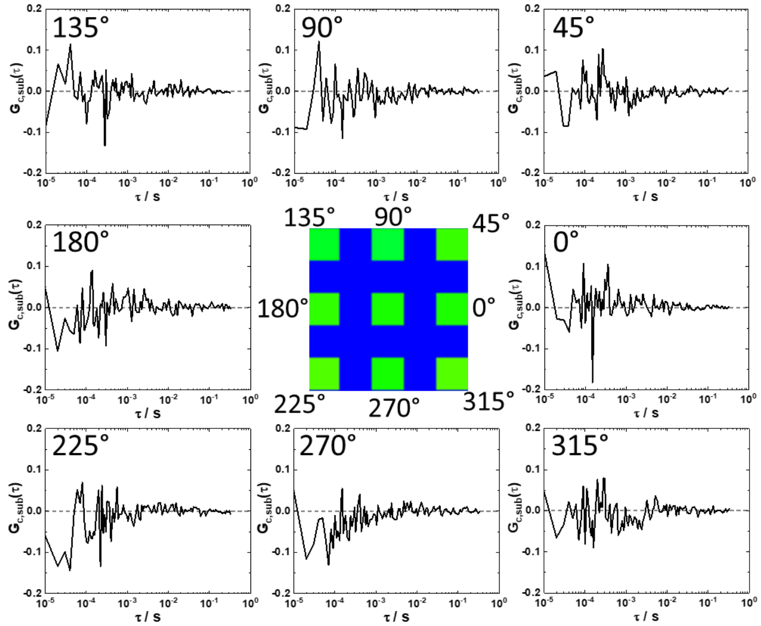

Figure S9. eGFP motion in a diluted aqueous solution assessed by two-foci cross-correlation analysis. Subtracted cross-correlation curves $G_{c,sub}(\tau)$ are calculated by subtracting one tfCCC calculated between two pixels from the other calculated in the opposite direction. For all lag time values, the amplitude of $G_{c,sub}(\tau)$ is close to zero, and the characteristic narrow peak that is typical when directed motion, *i.e.* flow is observed is not seen, as expected for free 3D diffusion of eGFP.

Finally, we show that while directed motion can also be observed in ACCs by residuals analysis, two-foci cross-correlation analysis is more powerful in this regard (Fig. S10).

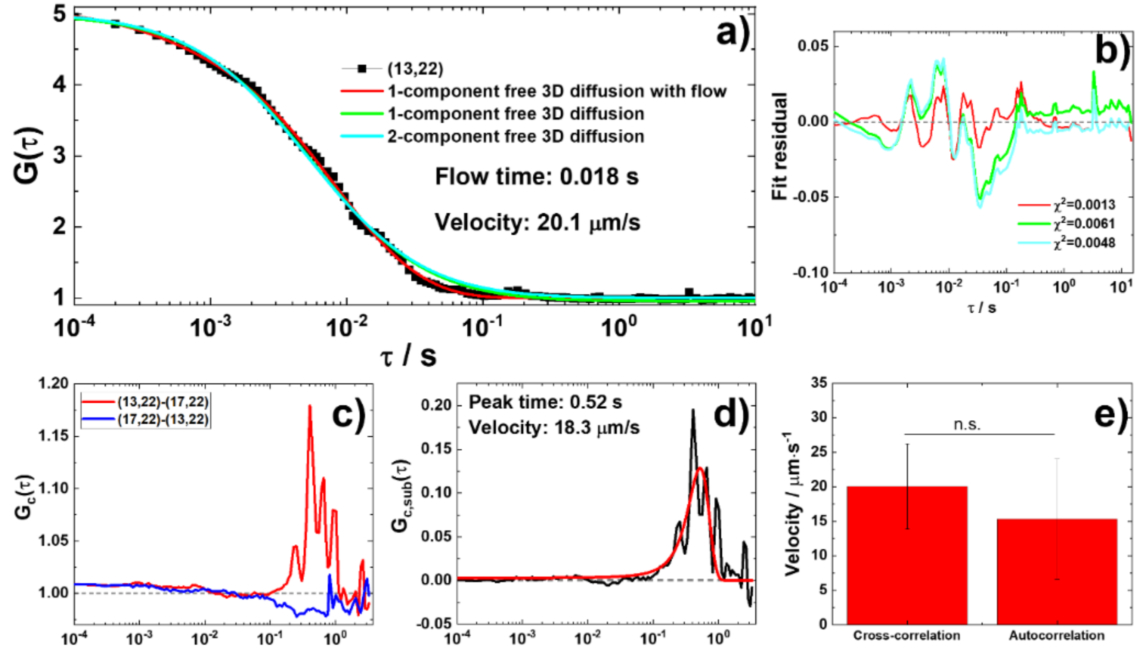

Figure S10. Autocorrelation and two-foci cross-correlation analysis of directional particle motion in a diluted suspension of 100 nm fluospheres. (a) Temporal autocorrelation curve in a single pixel (black) fitted to different model functions: free 3D diffusion of one component with flow (red), yielding a diffusion time τ_D = 5.5 ms and flow velocity of 20.1 μm/s; free 3D diffusion of one component without flow (green), yielding a diffusion time τ_D = 5.5 ms; and free 3D diffusion of two components (cyan), yielding two components with nearly equal contributions and roughly the same diffusion time τ_{D1} = 5.2 ms and τ_{D2} = 5.4 ms. **(b)** Unweighted fit residuals showing the difference between the experimental ACC and the values obtained by fitting different model functions indicated in a). The colour code is the same as in (a). Based on the analysis of fitting residuals, the best fit with the experimental ACC is obtained when using a model for free 3D diffusion with flow (red). **(c)** Two-foci cross-correlation curves (tfCCC) calculated in opposite directions, $G_{(13,22)\rightarrow(17,22)}(\tau)$ in the direction (13,22) → (17,22) (red) and $G_{(17,22)\rightarrow(13,22)}(\tau)$ in the direction (17,22) → (13,22) (blue). **(d)** The subtracted cross-correlation curve $G_{c,sub}(\tau) = G_{(13,22)\rightarrow(17,22)}(\tau) - G_{(17,22)\rightarrow(13,22)}(\tau)$ obtained after subtracting the tfCCCs calculated for different directions of motion (black) fitted using a Gaussian distribution function (red), yielding a peak at the lag time τ_{Peak} = 0.52 s, which is also the transit time between the two foci. Consequently, the 2D flow velocity could be assessed, v_{flow} = 18.3 μm/s. **(e)** Flow velocities determined by tfCCC analysis agreed well with values estimated by temporal ACC fitting using the model function for free 3D diffusion of one component with flow.

In Fig. S10, the following analytical functions were fitted to the experimentally derived ACCs.

For free 3D diffusion of one or two components, eq. (S8) was used:

$$G(\tau) = G(\infty) + \frac{1}{N}\cdot\left[1 + \frac{T}{1-T}e^{-\frac{\tau}{\tau_T}}\right]\cdot \left(\sum_{k=1}^{n}\frac{f_{D,k}}{\left(1+\frac{\tau}{\tau_{D_k}}\right)\sqrt{1+\frac{\omega_{xy}^2}{\omega_z^2}\cdot\frac{\tau}{\tau_{D_k}}}}\right) \qquad (S8)$$

where, $G(\infty)$ denotes the value to which the autocorrelation curve converges at infinitely long measurement time; T is the fraction of molecules in the triplet state and τ_T is the average relaxation time of the triplet state (when not applicable, T = 0); n is the number of freely diffusing components (n = 1 or 2); $f_{D,k}$ is the relative molar fraction of the k-th component (the sum of relative molar fractions is equal to 1); $\tau_{D,k}$ is the is the translational diffusion time of k-th component; ω_{xy} and ω_z are the $1/e^2$ radial and axial radii of the observation volume element (OVE), respectively. The ω_{xy} and ω_z are determined by mpFCS instrument calibration using a dilute suspension of 100 nm fluorospheres.

For free 3D diffusion of one component with flow. eq. (S9) was used:

$$G(\tau) = G(\infty) + \frac{1}{N}\cdot\left[1 + \frac{T}{1-T}e^{-\frac{\tau}{\tau_T}}\right]\cdot \frac{1}{\left(1+\frac{\tau}{\tau_D}\right)\sqrt{1+\frac{\omega_{xy}^2}{\omega_z^2}\cdot\frac{\tau}{\tau_D}}}\cdot exp\left[-\frac{\left(\frac{\tau}{\tau_f}\right)^2}{1+\frac{\tau}{\tau_D}}\right] \qquad (S9)$$

where τ_f is the transport time; $v_{flow} = \omega_{xy}/\tau_f$ is the flow velocity.

SI5: Glucocorticoid receptor nucleocytoplasmic translocation in live cells.

SI5a: Experimental data replicates and fitting

Fig. S11a shows 13 individual $G_{c,sub}(\tau)$ calculated at all positions shown in Fig. 3. Nuclear import (red) was observed at positions #1, 2, 3, 8, 9, 12 and 13, and nuclear export (blue) was observed at positions #4, 5, 6 and 11. In Fig. 11b, $G^{av}_{c,sub}(\tau)$ show average of nuclear import (red) and nuclear export (blue) in three different cells (Fig. 11b1-b3).

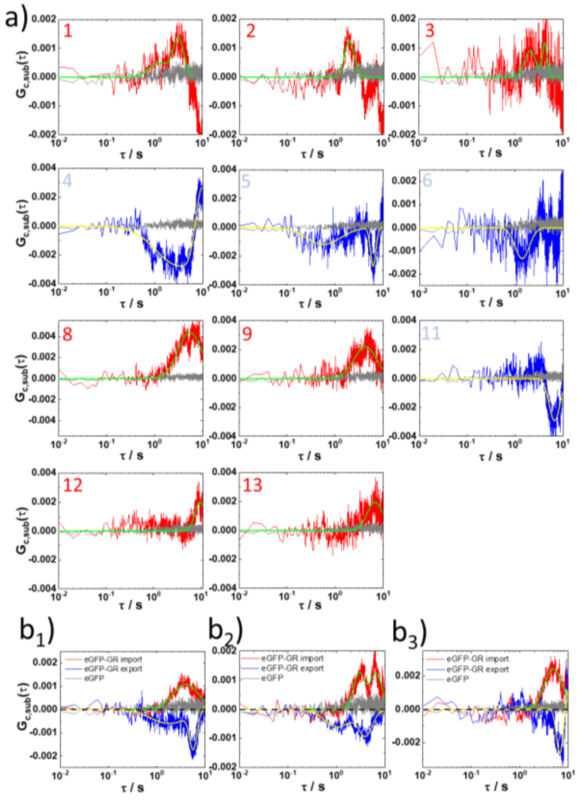

Figure S11. **Glucocorticoid receptor nucleoplasmic translocation in live cells. (a)** Individual $G_{c,sub}(\tau)$ showing nuclear import (red) and export (blue) at all sites shown in Fig. 3. Translocation time is determined by Gaussian distribution fitting to the $G_{c,sub}(\tau)$ (green/yellow). **(b1-b3)** $G^{av}_{c,sub}(\tau)$ show average of nuclear import (red) and export (blue) in three different cells. Data shown in b1 correspond to data shown in Fig. 3.

The Origin Multiple Peak Fit tool was used to fit the peaks in $G_{c,sub}(\tau)$ with Gauss peak function:

$$G_{c,sub}(\tau) = G_0 + \sum_{k=1}^{n} \frac{S_k}{w_k \cdot \sqrt{\frac{\pi}{2}}} \cdot e^{\left(\frac{-2\cdot(\tau - t_k)^2}{w_k^2}\right)} \quad \text{(S10)}$$

In eq.(S10), $G(\infty)$ denotes the value to which the cross-correlation curve converges at infinitely long measurement time; S, w and t are the area, width, and peak time of the fitted Gaussian model, respectively; n is the number of components, set as 1 or 2.

The average transporting time, t_{av} is computed when the subtracted cross-correlation shows multiple peaks:

$$t_{av} = \frac{s_1}{s_1+s_2} \cdot t_1 + \frac{s_2}{s_1+s_2} \cdot t_2 \quad \text{(S11)}$$

In case of single component, S_2 is equal to 0.

The translocation net rate, ψ_{net}, is calculated at each position in a live cell, as:

$$\psi_{net} = \frac{A_1}{t_1} + \frac{A_2}{t_2} \quad \text{(S12)}$$

In eq. (12), A_1 and A_2 are the amplitudes of the Gaussian distribution of each component. When a single peak was observed, A_2 is equal to 0.

The goodness of fit was assessed by residuals analysis (data not shown). When the residuals appeared to be ran-domly scattered around zero, we have regarded that the model describes the data well.

SI5b: Theoretical validation of net rate calculation

To corroborate the experimental analysis for net rate determination by $G_{c,sub}(\tau)$, numerical simulations were used to emulate the bidirectional movement of eGFP-GR into/out of the cell nucleus. As a first approximation, we have regarded in numerical simulations the passing of a molecule through the nuclear pore complex as a one-dimensional translation motion (Fig. S12).

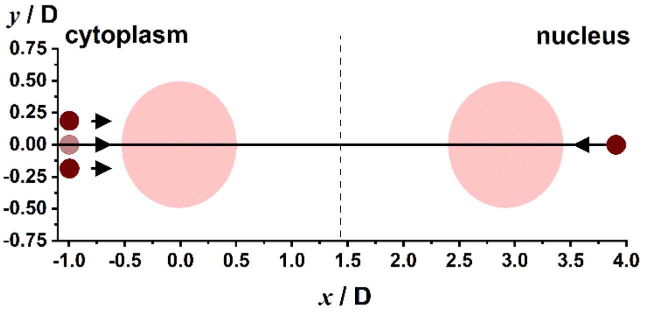

Figure S12. Schematic drawing of bidirectional translation of fluorescent particles along the *x*-axis through two adjacent observation volume elements (OVEs). The small wine circles represent fluorescent nanoparticles of diameter d = 100 nm. The large pink circles represent adjacent OVEs, the diameter of which was set in the numerical simulations to the experimentally determined value, D_{OVE} = 560 nm. The distance between OVE centres, Δl = 100 μm/63 = 1.59 μm, corresponds to real properties of our experimental setup – pitch distance between SPADs in the SPC[2] camera is 100μm and the microscope objective magnification is 63×. When simultaneous movement of two fluorescent particles is considered, their displacement from the central axis was D/10. The starting position of fluorescent particles from either side of the OVEs was arbitrarily set to $\Delta l/6$.

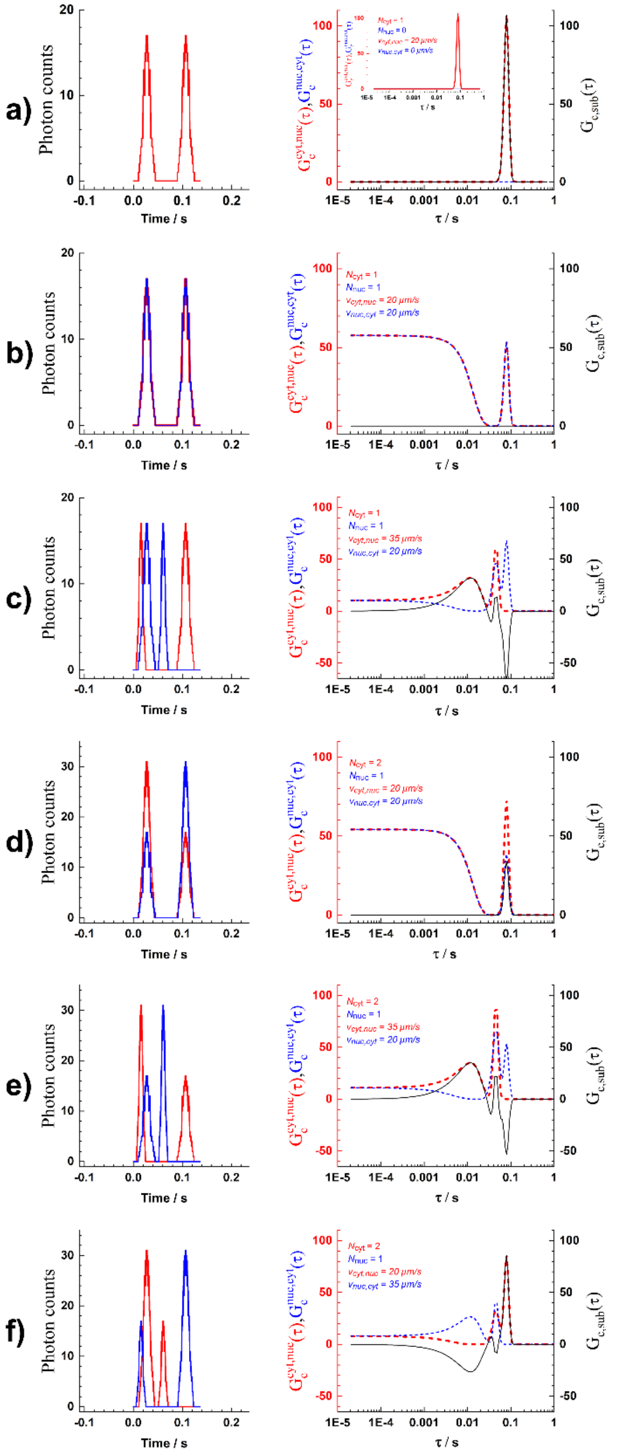

Figure S13. Numerical simulation of nuclear import/export. *Left panel*: Photon counts distribution in the cytoplasmic OVE (red) and the nuclear OVE (blue). *Right panel:* Corresponding tfCCCs $G_c^{cyt,nuc}(\tau)$ (red), $G_c^{nuc,cyt}(\tau)$ (blue), and $G_{c,sub}(\tau)$ (black). **(a)** N_{cyt} = 1, N_{nuc} = 0, $v_{cyt,nuc}$= 20 µm/s, $v_{nuc,cyt}$= 0 µm/s; **(b)** N_{cyt} = 1, N_{nuc} = 1, $v_{cyt,nuc}$ = 20 µm/s, $v_{nuc,cyt}$= 20 µm/s; **(c)** N_{cyt} = 1, N_{nuc} = 1, $v_{cyt,nuc}$= 35 µm/s, $v_{nuc,cyt}$= 20 µm/s; **(d)** N_{cyt} = 2, N_{nuc} = 1, $v_{cyt,nuc}$= 20 µm/s, $v_{nuc,cyt}$= 20 µm/s; **(e)** N_{cyt} = 2, N_{nuc} = 1, $v_{cyt,nuc}$= 35 µm/s, $v_{nuc,cyt}$= 20 µm/s; **(f)** N_{cyt} = 2, N_{nuc} = 1, $v_{cyt,nuc}$= 20 µm/s, $v_{nuc,cyt}$= 35 µm/s.

The results of numerical simulations are summarised in Fig. S13, with photon counts shown on the left and the corresponding tfCCCs to the right. In particular, the following distinct cases were considered: one fluorescent particle moving from the cytoplasm to the nucleus (Fig. S13 a); concomitant movement of two fluorescent particles, one from the cytoplasm and one from the nucleus, with the particles moving at the same (Fig. S13b) or different (Fig. S13c) speeds; and concomitant movement of three fluorescent particles, two fluorescent particles from the cytoplasm to the nucleus and one from the nucleus to the cytoplasm, with the particles moving at the same (Fig. S13d) or different (Fig. S13 e and f) speeds.

In the numerical simulations, photon counts distribution is emulated as the fluorescent particles (small, wine circles in Fig. S12) pass through the OVEs (large, shaded circles in Fig. S12). The excitation intensity distribution in the OVEs is Gaussian, and the fluorescence intensity, *i.e.*, photon count is zero when there is no fluorescent particle in the OVE (no background and no noise). The OVE diameter was set to the experimentally determined value, D_{OVE} = 560 nm, and the distance between the centres of the OVEs, Δl = 100 µm/63 = 1.59 µm, corresponds to real properties of our experimental setup – pitch distance between SPADs in the SPC^2 camera is 100µm and the microscope objective magnification is 63×. The time frame duration Δt = 20.74 µs, as in the real experiment. The fluorescent particle diameter was arbitrarily set to d = 100 nm. In cases when the movement of one or bidirectional motion of two fluorescent particles is considered, movement along the x-axis is simulated (Fig. S12, y/D = 0) and the obtained photon counts distributions are shown in Fig. S13 a, b and c, left panel. In cases when concomitant motion of three particles is considered, movement along a trajectory that is parallel to the x-axis (Fig. S12, y/D = 0.1) is simulated for the side from which two particles are moving and movement along the x-axis (Fig. S12, y/D = 0) is considered from the side from which one fluorescent particle is moving. The photon counts distributions for the latter case are shown in Fig. S13 d, e and f, left panel. The tfCCCs are calculated from the simulated photon counts distribution (Fig. S13, left panel) using eq. (2). Corresponding tfCCCs $G_c^{cyt,nuc}(\tau)$ and $G_c^{nuc,cyt}(\tau)$, as well as $G_{c,sub}(\tau)$, are shown in Fig. S13, right panel.

Of note, the results shown in Fig. S13 represent a small subset of many possible solutions of this complex problem. Nevertheless, they give us the first rough answers on how $G_{c,sub}(\tau)$ changes when the concentration of translocating molecules, their translation velocities, and the relative positions of nuclear pore complexes within the OVE are varied.

SI5c: Theoretical analysis of the effect of diffusion on the net rate of nuclear translocation

To emulate the effect of diffusion on the translation motion, we have numerically simulated the simplest case of combined translational and diffusion motion of a single fluorescent particle through two volume elements (Fig. S14).

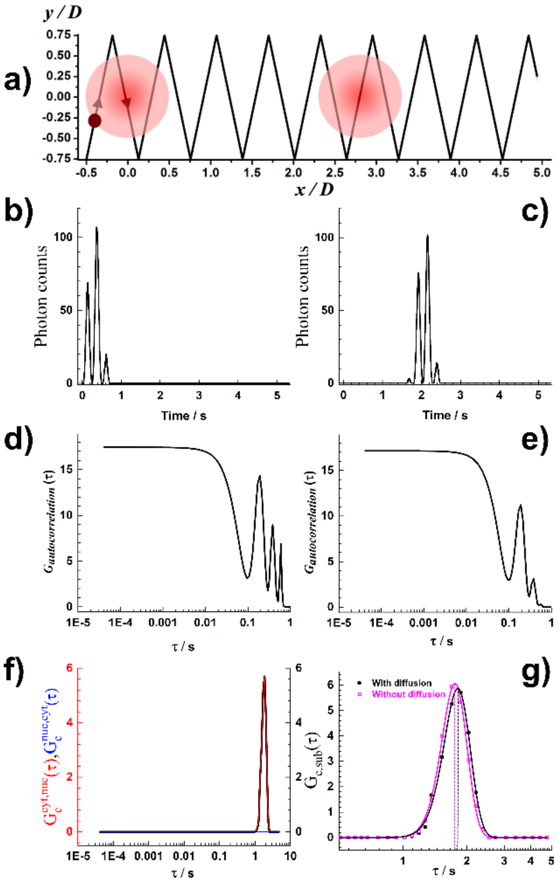

Figure S14. Emulating the effect of diffusion on the translation of molecules using tfCCC analysis. (a) Schematic drawing of the combined translational and diffusion motion of a single fluorescent particle (small wine circle) through two OVEs (large circles). **(b, c)** The simulated photon counts over time as the particle is passing through the OVE on the left (b) and right (c). **(d, e)** Autocorrelation curves reflecting fluorescent particle passage through the left and right OVE, $G_l(\tau)$ (d) and $G_r(\tau)$ (e), respectively. **(f)** The tfCCCs from left to right (red) and from right to left OVE (blue) and their difference, $G_{c,sub}(\tau)$ (black). **(g)** Diffusion broadens somewhat $G_{c,sub}(\tau)$, as reflected by $FWHM_m^{diff}$ = 0.65 s as compared to $FWHM_m$ = 0.59 s, and slightly changes the peak position towards longer lag times.

In the numerical simulations, the effect of diffusion on the directed motion was modelled as displacement in the *y*-axis direction – the translational velocity along *x*-axis is v_{transl} = 0.9 μm/s, and the *y*-component of the velocity changes over time the sign, but the magnitude of change is the same, v_{diff} = 4.3 μm/s (Fig. S14a). The fluorescent particle (Fig. S14a, wine circle) first passes through the left OVE, positioned in the cytoplasm, and after some time through the OVE on the right, positioned in the cell nucleus. The photon counts distribution over time reflecting fluorescent particle passage through the left, *i.e.*, cytoplasmic OVE, and the right, *i.e.*, nuclear OVE, are shown in FigS14 b and c, respectively. The corresponding ACCs $G_{cyt}(\tau)$ and $G_r(\tau)$ are presented in Fig. S14 d and e, respectively. The translational motion of the fluorescent particle can be clearly observed from the subtracted cross-correlation curve, $G_{c,sub}(\tau)$ = $G_{cyt,nuc}(\tau)$ - $G_{nuc,cyt}(\tau)$ (Fig. S14f). The $G_{c,sub}(\tau)$ peak is observed at a lag time τ_m^{diff} = 1.81 s, which corresponds to the time of flight between the two volume elements due to translational motion coupled with free diffusion. As expected, in the absence of diffusion, the $G_{c,sub}(\tau)$ peak is shifted towards slightly shorter lag times,τ_m = 1.75 s and a small peak broadening is noted in the presence of free diffusion, as reflected by the full width at half maximum, $FWHM_m^{diff}$ = 0.65 s as compared to $FWHM_m$ = 0.59 s (Fig. S14g).

References

[1] Krmpot, A.J.; Nikolić, S.N.; Oasa, S.; Papadopoulos, D.K.; Vitali, M.; Oura, M.; Mikuni, S.; Thyberg, P.; Tisa, S.; Kinjo, M.; Nilsson, L.; Terenius, L.; Rigler, R.; Vukojević, V. Functional Fluorescence Microscopy Imaging: Quantitative Scanning-Free Confocal Fluorescence Microscopy for the Characterization of Fast Dynamic Processes in Live Cells. Anal Chem. 2019, 91, 11129-11137.
